# Ensemble of coupling forms and networks among brain rhythms as function of states and cognition

**DOI:** 10.1038/s42003-022-03017-4

**Published:** 2022-01-21

**Authors:** Bolun Chen, Luis F. Ciria, Congtai Hu, Plamen Ch. Ivanov

**Affiliations:** 1 Keck Laboratory for Network Physiology, Department of Physics, Boston University, Boston, MA 02215 USA; 2 Mind, Brain and Behaviour Research Center, Department of Experimental Psychology, Faculty of Psychology, University of Granada, Campus de la Cartuja, Granada, 18071 Spain; 3 Division of Sleep Medicine, Brigham and Women’s Hospital, Harvard Medical School, Boston, MA 02115 USA; 4 Institute of Biophysics and Biomedical Engineering, Bulgarian Academy of Sciences, Acad. Georgi Bonchev Str. Block 21, Sofia, 1113 Bulgaria

**Keywords:** Neurophysiology, Network models

## Abstract

The current paradigm in brain research focuses on individual brain rhythms, their spatiotemporal organization, and specific pairwise interactions in association with physiological states, cognitive functions, and pathological conditions. Here we propose a conceptually different approach to understanding physiologic function as emerging behavior from communications among distinct brain rhythms. We hypothesize that all brain rhythms coordinate as a network to generate states and facilitate functions. We analyze healthy subjects during rest, exercise, and cognitive tasks and show that synchronous modulation in the micro-architecture of brain rhythms mediates their cross-communications. We discover that brain rhythms interact through an ensemble of coupling forms, universally observed across cortical areas, uniquely defining each physiological state. We demonstrate that a dynamic network regulates the collective behavior of brain rhythms and that network topology and links strength hierarchically reorganize with transitions across states, indicating that brain-rhythm interactions play an essential role in generating physiological states and cognition.

## Abstract (web summary)

Chen et al. analyze healthy human subjects during rest, exercise and cognitive tasks to show that synchronous modulation in the micro-architecture of brain rhythms mediates their cross-communications. They demonstrate that a dynamic network regulates the brain rhythm behaviour and that brain-rhythm interactions play an essential role in generating physiological states and cognition.

## Introduction

The human brain is an interconnected system where firing patterns of neurons integrate through networks of neuronal populations in a coordinated manner to facilitate communications between brain regions necessary for physiologic functions. At the system level, collective neuronal activity is manifested as brain waves and cortical rhythms^1^, with complex intermittent dynamics and organization across spatio-temporal scales^2–4^. Brain research has focused on the neuronal origins, spatial distribution, and temporal dynamics of brain waves^5–7^, and their role in facilitating physiological functions^8–11^. Robust associations were established between distinct brain rhythms and specific physiological states, neurophysiological and cognitive functions^12,13^, and clinical conditions^14,15^. Low-frequency (high-amplitude) delta waves, synchronized across cortical areas, dominate during deep sleep^9^. In contrast, high-frequency (low-amplitude) localized alpha waves are prevalent during quiet wakefulness and rest, while the amplitude and density of theta rhythms increase with the transition from wake to sleep^16^. Further, beta rhythms are a prominent signal of sensorimotor cortical activity during active wake and exercise^17^ with increasing power after exercise, while alpha rhythms (a marker of arousal) exhibit shift in power to higher frequencies following exhaustive exercise^18^ in response to change in body temperature, cerebral blood flow, and arousal level^19,20^. Moreover, alpha rhythms amplitude during rest is reduced by certain physiological states (eye opening, drowsiness) and cognitive functions (mental tasks)^9^, in contrast to gamma rhythms which exhibit elevated activity with affective and cognitive functions such as sensory perception^21^, attention^22^, decision making^23^ and memory formation^24^. Reciprocal activation of brain rhythms is also observed for cognitive functions with enhanced executive processing, where beta rhythms are suppressed before subjects’ response^25^ in association with motor preparatory processes^26^, while theta rhythms are enhanced following a correct response^25^ and diminish over error trials^27^. Cortical rhythms are also continuously modulated by cardio-respiratory autonomic function^28^, maturation, and aging^29^. Furthermore, pronounced difference in activation from opposite hemispheres or coexistence of pronounced brain rhythms responsible for competing physiological functions (e.g., alpha and delta waves) relate to pathological conditions^30^.

Thus, investigations focused on individual brain rhythms to understand their complexity and role in physiological states under health and disease. Dominant brain rhythms are traditionally considered signatures of physiological functions^8,9,11,16^—an approach motivated by empirical observations of quasi-steady-state behavior at large time scales during a given state, and gradual change in amplitude of brain rhythms with transitions across states^1,9^. In this classical paradigm, less attention is paid to non-dominant brain rhythms, how their dynamics impact the temporal and spatial organization of dominant rhythms, and whether interactions among dominant and non-dominant rhythms exhibit universal behaviors across brain areas that facilitate physiological functions.

However, brain rhythms stem from bursting activation in interconnected neuronal populations with heterogeneous functions controlled by feedback mechanisms and embedded in an extended network transcending brain regions^1,11,16,31^. Earlier studies employed amplitude modulation index, auto-correlations, scale-free, nonlinear, and criticality measures, focusing on dynamical characteristics of individual brain rhythms and their response to changes in states, conditions, and external/clinical perturbations across different time scales^3,32–35^. Other works have examined coupling and coherence of a given cortical rhythm across brain locations, extending this connectivity approach to different cortical rhythms separately^5,36–38^. Structural and functional connectivity studies have predominantly utilized fMRI data or cross-correlation on integrated EEG signals from different cortical locations^39–46^. In recent years, research groups have explored the interaction between cortical rhythms for selected pairs (e.g., *γ*-*α*, *γ*-*β*, *γ*-*θ*, *β*-*θ*) specifically in the context of working memory, perception, cognitive function, and consciousness^47–57^.

Here, we hypothesize that, due to neuronal network integration, both dominant and non-dominant cortical rhythms exhibit cross-communication that may be persistent across brain areas and unique for each physiological state. While recent studies have identified certain pairwise interactions^5,29,47–49,52,54–56,58^, the nature, functional forms of coupling, and how cortical rhythms interact as a network remain unknown. Thus, in addition to the traditional approach of defining states and functions through individual brain rhythms, we propose that coordinated interactions among all rhythms are essential for generating physiological states. Two scenarios could underlie such interactions, where (i) each state is associated with communication between some brain rhythms, or (ii) a dynamic network of interactions among all rhythms is a hallmark of physiological state and function. Further, coupling forms may be identical for all pairs of brain rhythms and specific for each state, or alternatively, states are characterized by a hierarchically structured network of brain-rhythm interactions with stronger or weaker network links representing different forms of pairwise coupling organized in distinct sub-networks.

We examine the micro-architecture in bursting dynamics of brain rhythms and probe their coupling through patterns of synchronous short timescale amplitude modulation on top of the quasi-steady behavior that dominates cortical dynamics over large time scales at each state. To identify forms of coupling between cortical rhythms, and how rhythms cross-communicate and collectively behave as a network, we develop a new analytic approach and an experimental protocol, where cortical dynamics of healthy subjects are studied during contrasting physiological states – quiet rest, vigorous physical exercise, and cognitive effort (Methods). High-intensity physical exercise after rest provides a reliable framework to study physiologic transitions since exercise is characterized by progressive physiological and cognitive adaptation^59^; cognitive tasks introduced after exercise allow to probe coupling forms and network interactions among brain rhythms when executive processing is enhanced^60^. The approach extends the analysis of single brain rhythms to probe time-evolving network interactions among multiple brain rhythms in response to change in physiological states.

We investigate the fundamental question of how cortical rhythms integrate as a network to generate and facilitate distinct physiological states and functions, and how these network interactions reorganize with transitions from one state to another. We analyze temporal patterns of reciprocal synchronous modulation in the spectral amplitudes of dominant and non-dominant cortical rhythms and their transient reorganization across states and uncover that a specific functional form characterizes the coupling profile for each pair of brain rhythms. Further, we find that each physiological state is uniquely defined by an ensemble of coupling profiles, and that all coupling forms fall in three major classes consistently observed at different brain areas and across subjects. Moreover, we demonstrate that brain rhythms continuously coordinate their dynamics and integrate as a network that undergoes complex hierarchical reorganization with transitions across states. The reported empirical observations demonstrate the necessity for departure from the traditional paradigm, where physiological states and functions are considered in association with specific brain rhythms, and underlie the importance of coordinated brain-wave interactions within specific network structure and network dynamics as a hallmark of physiological state and function.

## Results

### Synchronous amplitude modulation mediates brain-rhythm interactions

**Fig. 1:**
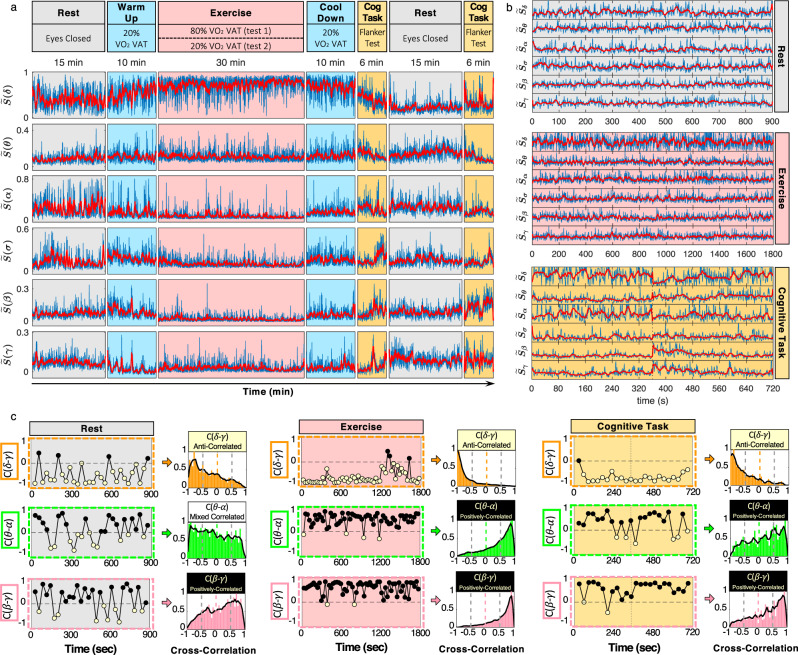
Synchronous modulation in spectral components of complex brain dynamics underlie distinct coupling profiles of brain-rhythm interactions. **a** (Top panel) Schematic diagram of the time course of experimental protocol consisting of consecutive sessions of distinct physiological states (rest, warm-up, exercise, cool-down, cognitive task) with corresponding experimental conditions and set-ups for physiological data recording. The protocol was repeated twice, for high and low physical effort during exercise, with respectively 80 and 20% ventilatory anaerobic threshold (VAT) (see Methods). (Bottom panels) Time series of the relative spectral power, $\widetilde{S}({{\Delta }}{f}_{i})$ of physiologically relevant brain rhythms (frequency bands, Δ*f*_*i*_ = {*δ*, *θ*, *α*, *σ*, *β*, *γ*}) derived from C3 EEG channel of a representative subject at 80% effort level (blue lines) and moving average (window 14 s; step 1 s; red lines) illustrating synchronous modulation in the micro-architecture of cortical rhythms within each physiological state (Methods). **b** Time series of relative spectral power $\widetilde{S}({{\Delta }}{f}_{i})$ from a representative subject for segments of rest, exercise (at 20% effort level), and cognitive tasks (blue lines; vertical dotted lines indicate the concatenation of two segments). Moving averages (red lines) show coordination among cortical rhythms during all three physiological states with synchronous modulation in $\widetilde{S}({{\Delta }}{f}_{i})$ at small time scales (s) indicating dynamic interactions between dominant and non-dominant brain rhythms. **c** Cross-correlation *C* between $\widetilde{S}({{\Delta }}{f}_{i})$ moving averages for selected pairs of brain rhythms in non-overlapping 30 s windows (left panels) and corresponding normalized histograms of cross-correlation values (right panels, population average) during rest, exercise (at 80% effort level), and cognitive task from the same representative subject. Note predominantly negative *C* values for *δ*-*γ* coupling and predominantly positive *C* values for *β*-*γ* coupling during all three physiological states (left panels in **c**). Correlation distribution profiles (right panels in **c**) reveal three classes of brain-rhythm coupling: stable anti-correlations (predominantly negative *C* values for *δ*-*γ* coupling); stable positive correlations (predominantly positive *C* values for *β*-*γ* coupling); and mixed correlations where the distribution profile changes from homogeneous during rest to positive cross-correlations during exercise and cognitive task (*θ*-*α* coupling).

To uncover effective interactions among brain rhythms, we perform time-frequency analysis on EEG signals at six cortical locations (Frontal: Fp1 and Fp2; Central: C3 and C4; Occipital: O1 and O2) for a group of healthy young subjects during rest, exercise, and cognitive task (Fig. 1a; Methods). At each cortical location, we study the spectral dynamics of distinct physiologically relevant brain rhythms (*δ*, *θ*, *α*, *σ*, *β*, *γ*) commonly used in neurophysiology, exercise physiology, cognitive psychology^1,11,15,16,61^. At large time scales of 10–30 mins, the EEG spectral dynamics exhibit quasi-steady-state behavior, characterized by the dominant presence of brain rhythms specific for each state (Fig. 1a), consistent with the traditional view that specific brain waves characterize physiological states and functions^9,12,62^. However, a close inspection of the relative spectral power of brain rhythms at short time scales of seconds reveals complex temporal patterns of coordinated bursts and synchronous amplitude modulation. Such continuous ‘ripples’ (red lines in Fig. 1b) on top of quasi-steady spectral dynamics associated with each physiological state stem from neuronal activities and encode brain-rhythm interactions. To quantify coupling, we compute cross-correlation for pairs of brain rhythms in short time windows of 30 s (Methods).

Analyses of EEG signals from C3 cortical area during experimental protocol sessions (physiological states) show interactions among brain rhythms manifested through synchronous amplitude modulation in their respective spectral power. These interactions lead to high degree of temporal cross-correlation, and exhibit distinct coupling forms for different pairs of brain waves (Fig. 1c)—e.g., during exercise, strong positive *β*-*γ* cross-correlation results from parallel increase or decrease in amplitude, while *δ*-*γ* coupling exhibits stable anti-correlation coupling (amplitude modulation in opposite directions). The diverse coupling forms demonstrate a remarkable complexity in brain-rhythm interactions.

**Fig. 2:**
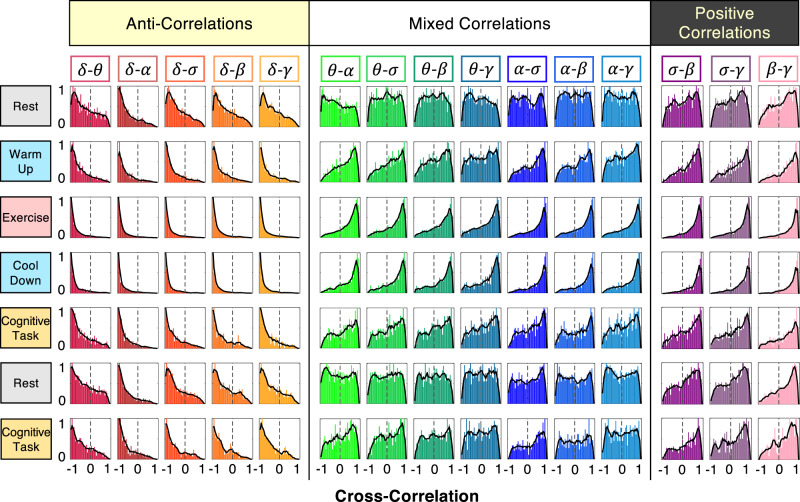
Transitions in brain-rhythm interactions across physiologic states. Cross-correlation distribution profiles for each pair of brain rhythms during different physiologic states (pooled data from all subjects in the database from two separate tests repeating the same protocol on different days at high (80% VAT) and low (20% VAT) level of physical effort during exercise; see also Supplementary Fig. 2). Distributions are rescaled by the peak values of cross-correlation and smoothed by moving average (solid lines, see Methods). For all states, interactions among distinct brain rhythms fall in three major classes: (i) anti-correlated coupling with predominantly negative correlation values for the pairs *δ*-*θ*, *δ*-*α*, *δ*-*σ*, *δ*-*β*, *δ*-*γ*; (ii) positively correlated coupling with predominantly positive correlation values for the pairs *α*-*σ*, *α*-*β*, *α*-*γ*, *σ*-*β*, *σ*-*γ*, *β*-*γ*; and (iii) mixed-correlated coupling for *θ*-*α*, *θ*-*σ*, *θ*-*β*, *θ*-*γ*, where the coupling profile gradually changes (along each column) from weakly negative to homogeneous and positive. Note that within these major classes, the coupling profile for each pair of cortical rhythms exhibits consistent modulation with transitions across states, e.g., fast-decaying tail during exercise (strong coupling), slow-decaying tail during the cognitive task (intermediate coupling), and fat tail during rest (weaker coupling). A unique ensemble of coupling profiles (along each row) characterizes each physiological state, and transitions across states are associated with changes in correlation profiles reflecting reorganization of brain-rhythm interactions. These findings indicate that a specific set of brain-rhythm coupling forms (an ‘alphabet’ of profiles) uniquely defines each physiologic state, and that a complex hierarchical organization in the cross-communication among brain rhythms is a hallmark of physiologic states and functions.

Next, we test whether brain-rhythm interactions are preserved with transitions from one physiological state to another. We find that certain pairs of brain rhythms show noticeably different coupling profiles for distinct physiological states (Fig. 1c)—e.g., *θ* and *α* rhythms are weakly anti-correlated during rest, become strongly positively correlated during exercise, and exhibit reduced positive correlations during a cognitive task. Such significant change in coupling with transitions across states unveil an intriguing, previously unrecognized association between brain-wave interactions and physiological states. These observations indicate that endogenous mechanisms of neuro-autonomic regulation influence coordinated activation among brain rhythms and raise the hypothesis that a specific set of coupling profiles representing all pairs of brain rhythms may uniquely represent each physiological state (Fig. 2).

### Distinct coupling profiles characterize interactions among brain rhythms

We quantify all pairwise interactions of brain rhythms and examine whether these interactions are consistent among subjects. During a given physiological state (rest and cognitive task), each pair of brain rhythms exhibits a distinct form for the distribution of cross-correlation values *C* (Fig. 2; Methods). Distribution profiles skewed to the left with a peak at *C* < 0 indicate strong anti-correlated coupling (predominant amplitude modulation in opposite directions) for the set of pairs *δ*-*θ*, *δ*-*α*, *δ*-*σ*, *δ*-*β*, and *δ*-*γ*. Distribution profiles skewed to the right with a peak at *C* > 0 show strong positive cross-correlation (predominantly parallel increase or decrease in amplitudes) for the set of pairs *σ*-*β*, *σ*-*γ*, and *β*-*γ*. For other pairs (*θ*-*α*, *θ*-*σ*, *θ*-*β*, *θ*-*γ*, *α*-*σ*, *α*-*β*, *α*-*γ*), the cross-correlation distributions are closer to uniform, where both positive and anti-correlated coupling is present in significant fractions of time, indicating a different mechanism of brain-rhythm coordination compared to the positive and anti-correlated pairs. These observations demonstrate surprisingly rich dynamics of diverse and transient nature in brain-rhythm interactions.

These observations reflect a complex temporal organization in brain-rhythm cross-communications that arise from collective dynamics of synchronous neuronal activity and is manifested by an ensemble of distinct coupling forms for different pairs of cortical rhythms: strong positive correlations with synchronous modulation in spectral power of cortical rhythms; strong negative correlations with anti-synchronous modulations; and more homogeneous coupling forms due to asynchronous neuronal activities leading to segments of on/off positive or anti-correlated coupling within a given physiological state (Fig. 2).

These complementary coupling relations among brain rhythms are observed for repeated test protocols (Supplementary Fig. 2) and in data from all subjects (Supplementary Fig. 7), demonstrating a universal behavior where each form of coupling plays a distinct role in regulating a given physiological state and function. This demonstrates the necessity to go beyond traditional approaches where physiological states are studied through dominant brain rhythms and their dynamics across brain areas^9^. In contrast, our results show that each physiological state is characterized by a set of coupling profiles (Fig. 2), resulting from transient on/off brain-rhythm interactions that are mediated through synchronous amplitude modulations in the micro-architecture of brain dynamics (Fig. 1).

### Unique ensemble of coupling profiles for each state

We find a robust association between the cross-correlation distribution profiles of brain-rhythm interactions and each physiological state (rows in Fig. 2). Moreover, the coupling profile for each pair of brain rhythms changes with transitions across physiological states (columns in Fig. 2): strong anti-correlated *δ*-*θ* coupling with pronounced negative peak and fast-decaying tail during exercise transitions into intermediate coupling with slower decaying tail during the cognitive task and to weaker negative coupling with fat tail during rest; strong positive *σ*-*β* coupling with pronounced positive peak and fast-decaying tail during exercise becomes intermediate with fat tail during a cognitive task and significantly diminishes during rest to more homogeneous coupling profile; *θ*-*α* coupling with strong positive cross-correlations during exercise changes to weaker positive coupling during cognitive task and weak anti-correlated coupling during rest.

Our analyses show that despite changes in brain rhythms coupling profiles with transitions across states, there is a global organization in the interactions among all brain rhythms that is present in all physiological states. Specifically, we identify three major classes of brain-rhythm interactions based on the evolution of cross-correlation profiles across states (Fig. 2): (i) anti-correlated pairs (*δ*-*θ*, *δ*-*α*, *δ*-*σ*, *δ*-*β*, *δ*-*γ*) that show stable negative correlations with pronounced peaks of cross-correlation consistently at *C* < 0; (ii) positively correlated pairs (*σ*-*β*, *σ*-*γ*, *β*-*γ*) that show stable positive correlations with cross-correlation peaks consistently at *C* > 0; (iii) mixed-correlated pairs (*θ*-*α*, *θ*-*σ*, *θ*-*β*, *θ*-*γ*, *α*-*σ*, *α*-*β*, *α*-*γ*) that exhibit state-dependent variations in the coupling, switching between negative and positive cross-correlations.

These three distinct classes of coupling profiles signify a hierarchical organization in brain-rhythm interactions and indicate a direct link between the coordinated activity of brain waves and the emergence of integrated physiological functions during rest, exercise, and cognitive tasks. Our observations demonstrate that intrinsic patterns of synchronous modulation in brain-wave amplitudes, embedded in the micro-architecture of EEG dynamics at short time scales (Fig. 1b), play an important role in mediating cross-communication among brain rhythms essential to generate physiological states. Notably, these interactions are masked when considering the absolute (instead of relative) spectral power in the frequency bands corresponding to different brain rhythms due to shifts in global EEG power at large time scales (see Statistical tests and Supplementary Fig. 8). These observations are consistent for repeated protocol sessions and all subjects (Supplementary Figs. 2 and 7), and reflect a robust intrinsic relation between interactions among brain rhythms and mechanisms of physiologic regulation, giving rise to an ensemble of robust coupling forms as a unique hallmark of state and function.

**Fig. 3:**
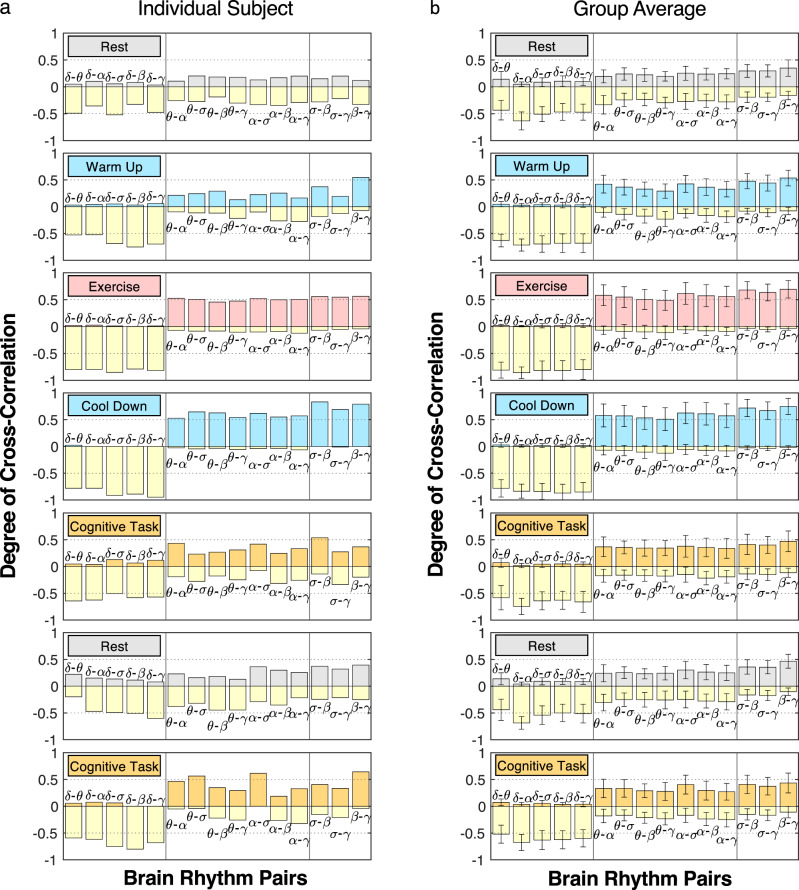
Robust stratification in the degree of coupling between brain rhythms across physiological states. Bar plots represent coupling strength derived from the cross-correlation distribution profile (Fig. 2) for each pair of brain rhythms at a given physiological state for (**a**) an individual subject and (**b**) the group average represented by pooled data from all subjects (Supplementary Fig. 3). For each pair of brain rhythms, bars height quantifies the probability of obtaining significant cross-correlation (∣*C*∣ > 0.5), where positive bars (colors matching states) represent the degree of positive correlation and negative bars (yellow color) represent the degree of anti-correlation (see Methods). Three classes of brain-rhythm interactions are observed across all physiological states: (i) strong anti-correlated interactions for pairs of rhythms *δ*-*θ*, *δ*-*α*, *δ*-*σ*, *δ*-*β*, *δ*-*γ*; (ii) strong positively correlated interactions for *σ*-*β*, *σ*-*γ*, *β*-*γ*; (iii) mixed cross-correlations for *θ*-*α*, *θ*-*σ*, *θ*-*β*, *θ*-*γ*, *α*-*σ*, *α*-*β*, *α*-*γ*, with a high degree of positive coupling during warm-up, exercise and cool-down, and more expressed negative (anti-correlated) coupling during cognitive task and rest. With transitions across physiological states, a clear stratification pattern in the degree of coupling between brain rhythms is consistently observed for all subjects in the database. Error bars in (**b**) represent group standard deviation. Such robust stratification in coupling strength based on physiological states reveals a universal complex organization of interactions among brain rhythms across subjects.

To test the validity of our findings at the individual subject level, we have calculated distribution profiles for all subjects separately and found consistency among subjects for the coupling profiles of all pairs of cortical rhythms at each physiological state. Our finding that the coupling profiles fall into three basic classes of interaction is robust as it is consistently observed for each individual subject (Supplementary Fig. 7). For each pair of brain waves during a given physiological state, all individual subjects’ distributions collapse and conform to a single shape (coupling profile) with a 95% confidence level (Wilcoxon signed-rank test), indicating that the functional form of coupling for each pair of brain rhythms is universal for all subjects. Remarkably, this data collapse is consistently observed for all pairs of brain rhythms during rest, exercise, and cognitive task, indicating the presence of an ‘alphabet’ of brain-wave communications (an ensemble of distinct coupling profiles) that uniquely characterizes each state at the individual subject level. Moreover, our analyses show reorganization in coupling strengths for all pairs of brain rhythms with transitions across states (see next sub-section) that is consistently observed for all subjects (Fig. 3). This universality among subjects in the coupling forms of cortical rhythm interactions and their classification in association with distinct physiological states indicate the presence of previously unknown basic laws of cortical rhythms regulation.

### Degree of coupling in brain-rhythm interactions

To quantify the brain-wave coupling profiles in Fig. 2, we introduce a measure of the degree of cross-correlation for all pairs of brain rhythms. Utilizing surrogate tests on randomized data (see Statistical tests and Supplementary Fig. 9), we identify two significance thresholds *C*_0_ = ±0.5 for physiologically significant positive correlations (*C* > 0.5) and negative correlations (*C* < −0.5). In the histograms shown in Fig. 2, the number of counts for ∣*C*∣ > 0.5 measures the fraction of time in data recording during a protocol session when significant positive or negative cross-correlation between frequency bands is detected—coupling profiles in Fig. 2 represent histograms of cross-correlation values {*C*_*ij*_} in non-overlapping 30 s windows (Methods). The coupling profiles are quantified by the areas of two extreme parts under the distribution curve, which are numerically proportional to the probabilities of observing statistically significant positive (*C* > 0.5) and negative (*C* < −0.5) cross-correlations (Supplementary Fig. 3). Thus, we define the degree of cross-correlation, associated with the coupling profile for each pair of brain rhythms in Fig. 2, by two symmetric matrices *D*^±^ to categorize different classes of coupling profiles (Methods). For a given physiological state, the matrix elements ${D}_{ij}^{\pm }=P(| {C}_{ij}| \, > \, 0.5)$ are the probabilities of observing strong positive and strong anti-correlated coupling for a given pair *i*-*j* of brain rhythms. Matrix elements ${D}_{ij}^{\pm }$ are represented as bars in Fig. 3. The matrices *D*^±^ are calculated for all physiological states and protocol sessions (Supplementary Fig. 4) and all EEG channel locations representing different cortical areas in the left- and right-brain hemispheres (Supplementary Figs. 13–15).

Utilizing this matrix representation, we establish three major classes of brain-rhythm interactions, based on the relative magnitudes of the degree of positive and anti-correlated coupling, that are consistently observed across all physiological states (pairs of brain waves in different coupling classes are separated by vertical lines in each panel of Fig. 3): (i) strong positive coupling (*D*^+^ > *D*^−^) persistent for all states; (ii) strong anti-correlated coupling (*D*^+^ < *D*^−^) for all states; (iii) mixed coupling where the relative magnitude of the degree of positive (*D*^+^) and negative (*D*^−^) coupling switches across states. As subjects go through multiple states during the experimental protocol, this graphic representation illustrates a clear stratification and reorganization of brain-wave couplings, consistently observed for all individual subjects (Fig. 3). Further, the three major coupling classes and the stratification pattern in coupling strength remain robust in respect to variations in the cross-correlation threshold *C*_0_ for rest to exercise and cognitive task (Methods). These observations indicate that, in response to changes in physiologic regulation, brain rhythms collectively adjust their amplitudes and flexibly adapt their forms of interaction and coupling strength to facilitate basic states and functions.

To validate the physiological relevance and statistical significance of the patterns in coupling strength and major classes of brain-wave coupling profiles (Figs. 2 and 3), we conduct several surrogate tests. First, we analyze pairs of brain rhythms where each rhythm is taken from a different randomly selected subject during the same physiological state. The resulting uniform cross-correlation distribution profiles fail to differentiate pairs of brain rhythms, and show no stratification across states (Supplementary Fig. 11). This surrogate test confirms that the observed variety of coupling forms for different pairs of brain waves at a given state, the three major classes of brain-wave interactions, and the stratification in degree of coupling across states represent fundamental physiological interactions.

To test whether the observed classes of coupling profiles (Fig. 2) and stratification patterns in coupling strength across physiological states (Fig. 3) depend on cortical areas, we further analyze data from six EEG channels (Fp1, Fp2, C3, C4, O1, O2). We find that the three major classes of brain-wave interactions and their stratification across states are consistently present at the Frontal, Central, and Occipital areas in the ipsilateral hemisphere and between contralateral hemispheres (Supplementary Figs. 13–15).

**Fig. 4:**
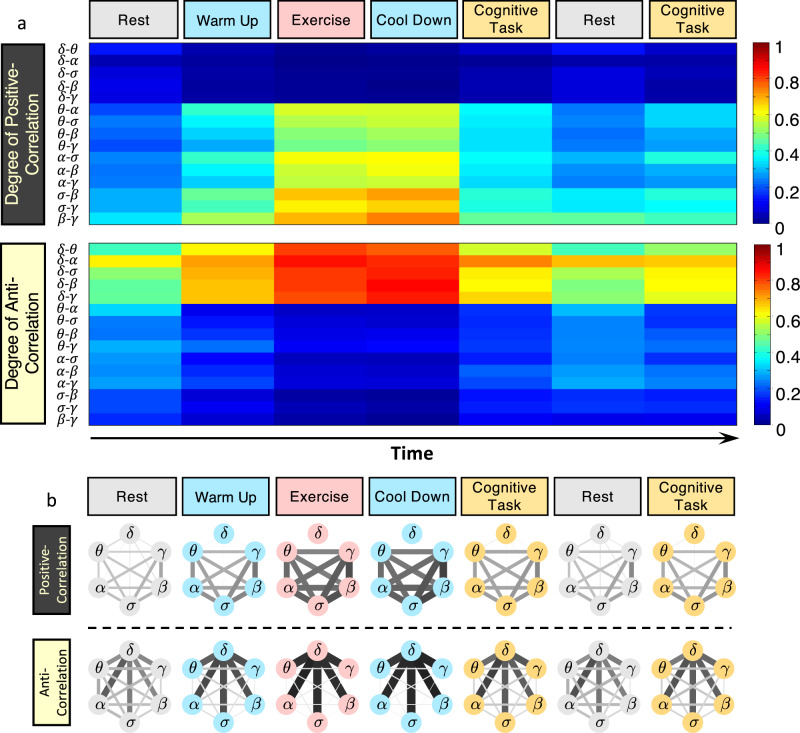
Network of cortical rhythm interactions and their evolution across physiological states. **a** Color-coded matrix representation of the degree of positive and anti-correlated coupling for all pairs of cortical rhythms shows the evolution of interactions in response to different physiological states. Increasingly synchronous modulation in the micro-architecture of bursting activity in *θ*, *α*, *σ*, *β* and *γ* rhythms leads to a high degree of positive coupling between these rhythms that is most pronounced during exercise (top panel, red color). This behavior is paralleled by asynchronous modulation in bursting dynamics in *δ* waves compared to other cortical rhythms leading to anti-correlated coupling most pronounced during exercise (red color), intermediate during the cognitive task (orange), and weak during rest. Note the consistency in coupling patterns (columns) for repeated segments of rest and the cognitive task where subjects are in quasi-stationary states. The difference between transient periods of warm-up and cool-down indicates a robust association of cortical rhythms coupling forms and strength with distinct physiological states. **b** Networks of brain-wave interactions for different physiological states, where network nodes represent cortical rhythms at the C3 EEG channel location, and links indicate the degree of coupling (line thickness and darkness correspond linearly to link strength). Coexisting sub-networks of positive (top panel) and anti-correlated (bottom panel) interactions among cortical rhythms with the specific topology of links strength uniquely define each physiological state. A pronounced network cluster of strong anti-correlated interactions of *δ* wave with all other brain rhythms is paralleled by a complementary cluster of positively coupled rhythms during exercise. With transitions to warm-up, cool-down, cognitive task and rest, these parallel networks of coordinated interactions evolve and are characterized by different topology and organization of links strength. Both positively and anti-correlated sub-networks of brain-rhythm interactions show transients where link strength can vary for different time segments within a given physiological state, reflecting complex dynamics of on/off brain-wave cross-communications are essential to facilitate flexibility and maintain the state. The coexistence of positive and anti-correlated interactions with different strengths among brain rhythms uniquely defines physiologic states. The specific topology and clustering of these networks for distinct physiological states demonstrate a direct association of the network cross-communication among brain rhythms with physiological states and functions.

Our approach of extracting brain-rhythm interactions from dynamical patterns of short-time synchronous modulation in brain-wave activation allows to identify and trace transient phenomena in each physiological state: When the relative spectral power of one frequency band (e.g., *δ* rhythm) abruptly changes in a short time window of several seconds, the relative spectral power of other rhythms (e.g., *θ*, *α*, *σ*, *β*, *γ*) instantaneously and reciprocally respond to this transient event, while maintaining relatively steady compared to each other. These coordinated responses reflect strong anti-correlation between *δ* and other waves, various degrees of stable positive correlation between *σ*, *β* and *γ* waves, and adjustable degrees of negative, positive and homogeneous forms of coupling depending on physiological states for the remaining pairs of brain waves—behavior which cannot be observed when brain-wave absolute spectral power is considered (Methods; Supplementary Fig. 8). Furthermore, such reciprocal coupling relations among pairs of brain rhythms are clearly manifested in the evolution of coupling strength across consecutive protocol sessions corresponding to distinct states (Fig. 4a). Mediated through synchronous brain-wave activation patterns embedded in the micro-architecture of brain dynamics on top of global trends in absolute spectral power of dominant and non-dominant brain waves (Fig. 1), such interactions play an essential role in maintaining a given physiological state and in facilitating transitions across states by continuous adjustments in coupling forms (Fig. 2) and coupling strength (Fig. 3), to adapt to internal and external perturbations.

Finally, the measure we introduce to quantify brain-wave interactions (Supplementary Fig. 3 and Fig. 3) represents the frequency of occurrence of transient coupling events, where for a fraction of time during a given state brain rhythm can be either positively or negatively coupled (positive and negative bars for each pair of waves in Fig. 3). Thus, our findings reveal a dual nature of brain-wave interactions, where each pair of brain rhythms adjusts the positive and negative components in the coupling to facilitate physiological functions at a given state and flexibility for transitions across states.

### Networks of brain-rhythm interactions

To investigate the complex behavior of integrated brain-wave communications, we construct networks of positive and anti-correlated interactions based on the established coupling forms (Fig. 2) and coupling strength (Fig. 3) for all pairs of brain rhythms and track the evolution of network structure with transitions from rest to exercise and cognitive task (Fig. 4a). The degree of coupling between brain waves can be presented as interaction matrices of two sub-networks, where brain rhythms are network notes and the matrix elements ${D}_{ij}^{\pm }$ represent the strength of network links (Methods). This network approach quantifies brain-rhythm coupling resulting from coexisting synchronous and anti-synchronous behaviors represented by two parallel sub-networks of positive and anti-correlated links. The dynamic network representation in Fig. 4b reflects the emerging collective behavior of coordinated brain-wave activities whose evolution across physiological states can be tracked in time as snapshots of distinct network configurations, and thus, demonstrates how brain waves at different cortical locations integrate as a network to generate physiological states.

During exercise, the *D*^−^ sub-network exhibits a pronounced clustering of anti-correlated interactions with very strong links between *δ* and all other cortical rhythms (bottom, Fig. 4b). We also identify a complementary *D*^+^ sub-network that is composed of positive links between all brain rhythms except *δ* during exercise (top, Fig. 4b). Thus, two major sub-network clusters of positive and anti-correlated interactions uniquely define the state of exercise. As subjects undergo transitions across warm-up, cool-down, cognitive task and rest, coupling strength in the cluster of anti-correlated links gradually decreases, while new anti-correlated interactions among brain waves emerge during warm-up and cool-down, and become more pronounced during cognitive task and rest. Notably, links between *θ*, *σ*, *α*, *β*, *γ* in the *D*^+^ sub-network, which are strongly positive during exercise, gradually decrease in strength during cognitive task and rest (top, Fig. 4b), and exhibit increased anti-correlated component (bottom, Fig. 4b). In contrast, in the process of these transitions, interactions of *δ* with other rhythms become increasingly positively correlated, leading to emerging new links. These findings reveal a competing nature of the positive and negative coupling component for each pair of brain rhythms, and demonstrate a complex reorganization in the network of brain-wave interactions to adapt to changes in physiologic conditions.

The complementary *D*^+^ and *D*^−^ sub-networks exhibit a similar pattern of change in network topology (i.e., reorganization in network connectivity and link strength) with transitions from rest to warm-up, exercise, cool-down and cognitive task, where links of both positive and anti-correlated coupling synchronously increase or decrease in strength (Fig. 4b). Such reorganization with transitions across states is observed globally at the network level as well as at each node, indicating a complex hierarchical cross-communication among brain rhythms. In particular, during rest *θ*-*β* interaction is characterized by a positive cross-correlation coupling component in the *D*^+^ matrix, the strength of which gradually increases during cognitive task and warm-up, and is highly expressed during exercise and cool-down (top, Fig. 4b). In contrast, the negative anti-correlated component in the *θ*-*β* coupling, which is intermediately strong during rest and cognitive task, significantly declines during warm-up, exercise, and cool-down (bottom, Fig. 4b). While in the traditional research framework *θ* and *β* waves are viewed as the respective predominant brain rhythms that are characteristics for cognition and physical activity, the reported here empirical findings provide new insight on the effective network interactions between these and other cortical rhythms as a hallmark of physiological state, where the complementary nature of positive and anti-correlated coupling plays a key role. In parallel to changes in *θ*-*β* interactions across states, *α*-*δ* coupling remains strongly anti-correlated during all states (bottom, Fig. 4b), while interactions of *α* with all other rhythms are characterized by weak anti-correlated links in the *D*^−^ sub-network (bottom, Fig. 4b) and reciprocally by strong positive links in the *D*^+^ sub-network (top, Fig. 4b) during physical activity (warm-up, exercise, and cool-down). In contrast, for cognitive tasks, *α* exhibits a comparable degree of positive and anti-correlated coupling (except the *α*-*δ* link), which implies a distinct role of *α* coupling compared to *θ*- and *β*-related interactions.

**Fig. 5:**
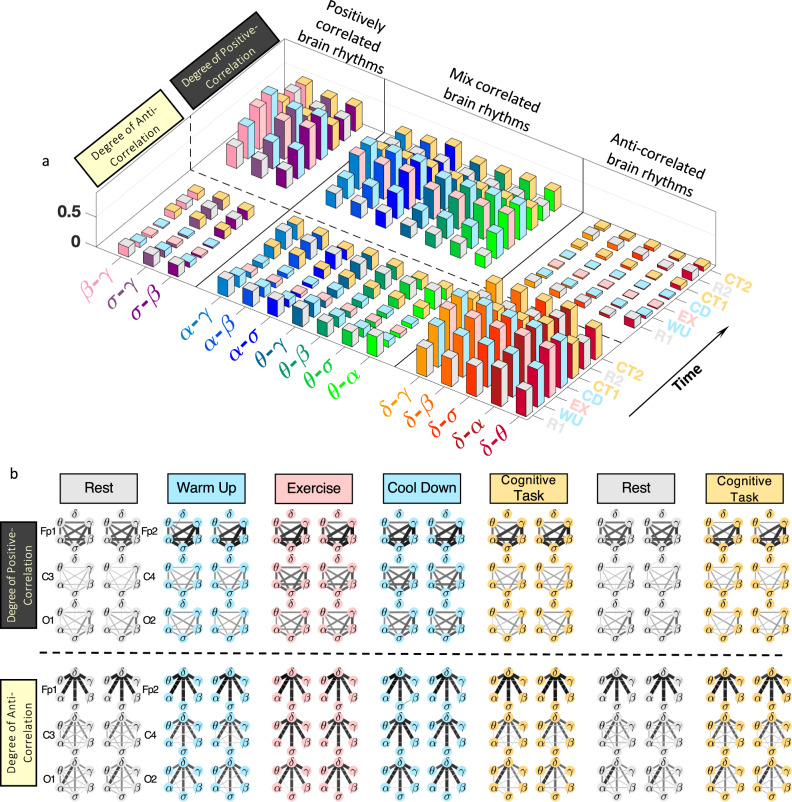
Network communications and topological clustering of brain-rhythm interactions at different cortical locations uniquely represent physiological states. **a** Three-dimensional representation of the degree of positive and anti-correlated components of coupling (bars height shows coupling strength for positive (*D*^+^) and anti-correlated (*D*^−^) coupling, Methods) for the three major classes of brain-rhythm interactions (positively correlated, mix correlated and anti-correlated pairs of brain rhythms; Fig. 2), and their evolution in time (Experimental protocol, Methods) across physiological states. The panel shows coupling for all pairs of brain rhythms at the C3 EEG channel location and provides a complete picture of the response in brain-rhythm interactions to changes in physiological states. **b** Networks representing brain-rhythm interactions at six cortical locations (Fp1, Fp2, C3, C4, O1, O2) for the left- and the right-brain hemispheres during different physiological states. Sub-networks of positive (top panel) and anti-correlated (bottom panel) components of brain rhythms coupling exhibit variations in topology and links strength organization depending on cortical locations and physiological states. Line thickness and darkness are scaled linearly to link strength. A clear symmetry in network structure is observed between the left (Fp1, C3, O1) and the right (Fp2, C4, O2) hemisphere for each physiological state. Sub-networks representing brain-wave interactions at the frontal areas are characterized by stronger links compared to the central and occipital areas, a behavior more pronounced at rest and cognitive tasks. Notably, despite their differences, networks at each cortical location undergo the same pattern of reorganization in topology and link strength across physiological states, indicating a global mechanism regulating brain-rhythm interactions at different cortical locations.

Next, we examine the configuration of network links considering separately the positive and anti-correlated coupling component of the each link. As subjects traverse through physiological states, a complex interplay emerges for positive and anti-correlated coupling components among brain rhythms (Fig. 5a)—(i) a cluster of network links for a subset of brain waves (*δ*-*γ*, *δ*-*β*, *δ*-*σ*, *δ*-*α*, *δ*-*θ*) characterized by dominant anti-correlated coupling component during all states (${D}_{ij}^{-} \, > \, {D}_{ij}^{+}$; high bars, bottom right corner in Fig. 5a); (ii) a group of brain waves (*β*-*γ*, *σ*-*γ*, *σ*-*β*) where network links are dominated by positive coupling component during all states (${D}_{ij}^{-} \, < \, {D}_{ij}^{+}$; high bars, top left corner in Fig. 5a); and (iii) brain waves with interactions mediated through varying degree of positive and anti-correlated coupling, where anti-correlated coupling dominates during rest, positive coupling dominates for warm-up, exercise and cool-down, and balanced degree of both anti-correlated and positive coupling during cognitive task.

Extending our analyses to different cortical areas, we find complex network organization of brain-rhythm interactions with pronounced clustering that is specific for each sub-network of positive and anti-correlated interactions: a cluster of strong positive links among *α*, *σ*, *β*, *γ* in the *D*^+^ sub-network and a cluster with strong anti-correlated links connecting *δ* with *θ*, *α*, *σ*, *β*, *γ* in the *D*^−^ sub-network (Fig. 5b). These topological clusters characterize brain-wave interactions at all cortical locations and are more pronounced at the Frontal areas with gradually declining links strength at the Central and Occipital areas where additional links emerge leading to higher connectivity—a robust behavior observed ipsilaterally for both *D*^+^ and *D*^−^ sub-networks across all subjects in all physiological states. Further, our analyses show a symmetry between left- and right-brain hemispheres that is consistently present in both sub-networks at all cortical areas and for all states (Fig. 5b).

Remarkably, with transitions across states, both sub-networks undergo complex reorganization in connectivity and links strength, where cluster-links are stronger during warm-up, exercise, and cool-down, while rest and cognitive tasks are characterized by higher network connectivity and weaker links (Fig. 5b). Notably, the same stratification in network structure (connectivity and links strength) across states is consistently observed in both sub-networks for all cortical areas in all subjects, indicating a robust mechanism of regulation underlying brain-rhythm network communications in association with each physiological state.

Our analyses show that all cortical rhythms (dominant and non-dominant) at a given cortical location continuously coordinate their dynamics and integrate as a network to facilitate brain function during distinct states. This coordination among cortical rhythms is mediated through a hierarchically structured network characterized by sub-networks and network motifs within each sub-network—e.g., *σ*-*β*-*γ* form a motif characterized by stronger links within the positively correlated (*D*^+^) sub-network (Fig. 5b) that is present at all cortical locations and across states. Based on the identified stratification of coupling forms (links strength) into three classes (Fig. 3), we find that at each state the network of interaction among all cortical rhythms consists of two coexisting sub-networks representing positive (synchronous) and anti-correlated (asynchronous) coupling, and that within each sub-network there are motifs of stronger and weaker links connecting sub-groups of cortical rhythms. Our findings demonstrate that both sub-networks and motifs within sub-networks change and reorganize with transitions across physiological states (Fig. 5), indicating hierarchical reorganization in cortical rhythms network interactions.

## Discussion

The classical paradigm of brain research addresses fundamental questions related to the origins of brain waves, their dynamics, and the role that individual dominant and non-dominant brain rhythms and their spatio-temporal organization across brain areas play in generating specific physiological states and functions^1,9^. Studies have traditionally focused on variations in spectral power of specific brain waves and their coherence across brain areas^63–65^, with limited investigations on the synchronous occurrence of specific pairs of cortical rhythms^49,54,58,66^, mainly in the context of memory and cognition^29,67,68^. To test the hypothesis that physiological states cannot be fully described by focusing on individual brain rhythms and isolated pairwise interactions, we systematically study interactions among all physiologically relevant cortical rhythms. We discover that distinct coupling forms and a dynamic network with hierarchical structure characterize interactions between dominant and non-dominant brain rhythms that uniquely define each physiological state and function.

Probing the micro-architecture of cortical dynamics, we demonstrate that transient epochs of synchronous and asynchronous brain-wave modulation at short time scales (Fig. 1b), traditionally regarded as bursts and noisy fluctuations embedded in the quasi-steady trends of spectral dynamics at large time scales, carry essential information about the nature of brain-rhythm interactions and physiological states. Our analyses of temporal patterns in the amplitude of brain rhythms activation derived from EEG data in healthy young subjects during repeated periods of quiet rest, moderate–high-intensity aerobic exercise, and cognitive function (Eriksen flanker task) (Fig. 1a; Methods), show that continuous coordination among all cortical rhythms (*δ*, *θ*, *α*, *σ*, *β*, *γ*) underlies each physiological state (Fig. 1b). We empirically identify functional forms of cortical rhythms coupling, and we discover that a specific cross-correlation distribution profile characterizes synchronous and asynchronous patterns in amplitude modulation that mediate the interaction for each pair of brain rhythms (Fig. 1c). Further, we find that different pairs of brain rhythms are characterized by distinct coupling forms (Fig. 2) and the strength of coupling (Fig. 3). Such diversity in the forms of cross-communication is needed to maintain flexibility in coordination among pairs of brain rhythms, and to facilitate emerging collective behaviors associated with various physiological states and functions.

Our investigations demonstrate that despite the diversity in brain-rhythm interaction patterns, all coupling forms fall in three major classes, where some pairs of rhythms maintain the same form of positive and anti-correlated coupling during all states, while other pairs are characterized by mixed-correlated interactions with different coupling forms for different states (Fig. 2). The collective behavior of brain rhythms is represented by an entire ensemble of coupling profiles that is consistent for all healthy subjects and uniquely defines each physiological state (Fig. 2). This ensemble of profiles captures the dynamics of reciprocal amplitude modulation in brain rhythms, and demonstrates a complex transient nature of brain-wave interactions at a given physiological state. Remarkably, while the functional forms of brain-rhythm coupling remain structured within three major classes, the coupling strength changes with transitions across states, leading to a hierarchical reorganization of the entire ensemble of cortical rhythm interactions (Figs. 2 and 3). Our observations that pairs of brain rhythms exhibit distinct functional forms of coupling, which coexist during a given physiological state and change with transitions across states, indicate a previously unrecognized complexity in the temporal organization of cortical rhythms, and demonstrate a robust association between coordinated cross-communication among brain rhythms and physiological states.

We develop a network approach (SANA method; section Methods) to examine the integrated behavior of brain rhythms, where network links represent the coupling strength (Fig. 3). We discover that each physiological state is characterized by a dynamic network of brain-rhythm interactions with the hierarchical organization of two coexisting sub-networks with the different topology of network links strength and motif organization, reflecting the degree of positive and anti-correlated coupling components for each pair of rhythms (Fig. 4). This hierarchical organization and clusters within sub-networks remain stable across ipsilateral cortical areas—network topology at the Frontal, Central, Occitipal areas is preserved, despite a gradual change in network links strength from the Frontal to the Occipital area (Fig. 5). Moreover, coupling forms, links strength, and network clusters are symmetric across the left- and right-brain hemispheres. These network characteristics are consistently observed for repeated segments of each physiological state within a protocol session and for repeated protocol sessions on different days (Methods) for all subjects, indicating universality in brain-rhythm interactions that must stem from an endogenous mechanism of physiologic regulation. Further, with transitions from one physiological state to another, brain-rhythm interactions exhibit reorganization in sub-networks cluster structure and link strength, indicating that continuous coordination in the dynamics and coupling of all brain rhythms and their integration as a network is a fundamental signature of physiological state and function.

In the context of rest, exercise and cognitive task, the reported here results indicate that in addition to the traditional framework, where spatio-temporal characteristics of a specific (pronounced, dominant) brain-rhythm are associated with each state – e.g., *α*-waves during quiet rest^9,18^, *β*-waves during exercise^17,25^ and *γ*-waves during cognitive task^8,11^ – the specific functional forms of coupling and hierarchical organization in network interactions among all cortical rhythms play an essential role for physiological states. Earlier research^59^ pointed to an overall increase of oscillatory brain activity during moderate–high-intensity exercise with respect to resting state, observed for all cortical rhythms and locations. Our results show that physical exercise as a highly demanding state where overall increase of oscillatory brain activity is paralleled by dynamic network interactions among cortical rhythms with increased links strength in certain network clusters (Figs. 4b and 5). The pronounced transition in topological clustering of brain-wave networks from resting state to moderate–high-intensity exercise indicates a direct association with states, where brain rhythms coordinate their activation and collectively adjust their interactions in response to changes in physiologic regulation due to physical exertion. The robustness of this association is further confirmed by our observations that network clusters and links strength recover their resting state configuration after vigorous exercise. Repeated segments of the same state within a protocol session and repeated experimental protocols with different levels of physical efforts during exercise (80 and 20% VAT, Methods) consistently show the same major classes of coupling forms, similar network structure, and reorganization with transitions across states (Supplementary Figs. 2 and 4). Notably, higher levels of physical effort (80% vs. 20% VAT) are associated with stronger brain-rhythm network interactions. Moreover, our finding that warm-up and cool-down segments before and after exercise (maintained at the same low-intensity effort of 20% VAT and same cadence of 60–90 rpm) are characterized by the same network topology but significantly stronger links during cool-down, indicates that brain-rhythm interactions are sensitive to fatigue (Fig. 4b). Cognitive tasks also alter the coupling forms and coordination among pairs of cortical rhythms, where sub-networks of positive and anti-correlated coupling exhibit stronger links compared to rest—a behavior consistently observed across subjects for repeated segments of cognitive tasks (Figs. 4 and 5, Supplementary Figs. 2 and 4). While subjects performed the cognitive task equally well, the observed sensitivity in network characteristics to enhance cognition opens perspectives for further investigations of how brain rhythms coordinate as a network to facilitate cognitive functions at different levels of accuracy, and to develop novel network-based markers that differentiate effects of accumulated physical effort and attention focus^69^.

The uncovered here probabilistic transient nature of brain-wave interactions, where periods of synchronous and asynchronous modulation switch on and off to mediate coexisting forms of coupling with varied strength, provides essential flexibility for brain rhythms with distinct characteristics to dynamically adapt and coordinate as a network in response to internal and external perturbations in the process of maintaining physiological functions during a given state and for facilitating transitions across states. These empirical findings provide new insights into the basic regulatory mechanisms of diverse physiological states and may guide future efforts to understand the role of microscopic signaling pathways and integration processes in neuronal populations dynamics play in regulating physiological functions^70^.

This is a preliminary, pilot study on a limited number of subjects with a pre-configured setup of only 30 EEG electrodes, and analyses of a larger cohort of subjects with a denser EEG electrodes setup may be needed to confirm the validity of the results. While our study is based on data from 19 subjects, the unique experimental protocol we developed combines consecutive and repeated sessions of distinct physiological states (rest, exercise, and cognitive task) within a single test. Thus, the availability of data from repeated sessions of the same state allows to test our hypothesis, and confirm the consistency of our findings that distinct coupling profiles and coordinated networks of cortical rhythm interactions characterize each state. Moreover, each participant repeated the entire experimental protocol on two separate days, effectively yielding 38 recordings in our analysis (Methods). Since each experimental protocol is ~90 min long (with continuous, 1000 Hz EEG recording), the size of the data set is comparable to transitional pilot (non-clinical) studies in the areas of cognitive neuroscience and exercise physiology where brain dynamics are studied.

To confirm that physiological states can be differentiated through the functional form and strength of coupling between cortical rhythms not only at the group average level but also at the individual subject level, we obtained for each subject separately the ensemble of coupling profiles for all pairs of cortical rhythms at all physiological states (Supplementary Fig. 7). For each pair of brain waves during a given state (row of panels in Supplementary Fig. 7), all individual subjects’ distributions conform to a common shape (coupling profile) with 95% confidence level (Wilcoxon signed-rank test), indicating that the functional form of coupling for each pair of brain rhythms is universal for all subjects. Remarkably, data collapse of the cross-correlation distribution profiles is consistently observed for all pairs of brain rhythms, indicating the presence of an ‘alphabet’ of brain-wave communications (an ensemble of coupling profiles) that uniquely characterizes each physiological state—a differentiation observed at the level of the individual subject. Supplementary Fig. 7 shows consistency of (i) coupling profiles for all pairs and classes of cortical rhythm interactions, and (ii) change in the functional form of coupling profiles with transitions across states for all individual subjects. Thus, the group average coupling profiles (Fig. 2) and network structure of brain rhythm interactions (Figs. 4b and 5b) have discriminative power for different states at individual subject’s level.

To our knowledge, the reported here observations present first empirical findings that (i) pairs of cortical rhythms communicate through distinct forms of coupling, (ii) coupling forms for all pairs of cortical rhythms fall within three major classes, and (iii) a specific network configuration of coupling strengths among all cortical rhythms uniquely defines basic physiological states such as rest, exercise and cognitive function. Currently, there is no theoretical framework and modeling approaches that account for these observations. The findings provide key information for future modeling efforts focusing on how neuronal populations that generate different cortical rhythms couple with each other; what are the temporal, functional, and spatial characteristics of feedback loops in the global neuronal network that accounts for synchronous and asynchronous modulation between cortical rhythms at different time scales and cortical locations; how transitions in physiological regulation across states affect the coupling form/strength of cortical rhythms; and the mechanisms through which states potentiate interactions of neurons within an assembly and neuronal populations across assemblies in different brain areas to generate specific synchronization networks among cortical rhythms.

Earlier studies of physiological systems (cardiac, respiratory, locomotor, brain) have focused on individual physiological variables and source signals (heart rate, respiratory intervals, gaits, EMG, and EEG data) to establish associations between dynamical features (auto-correlations, scaling measures, criticality, linearity/non-linearity) with different states (rest/exercise, sleep/wake, sleep stages, circadian phases, cognitive tasks, consciousness)^3,33,71–82^ and probe underlying control mechanisms^83–86^. In the context of brain dynamics, long-range correlations, auto-correlation window decay, and scaling behavior have been utilized to characterize intrinsic neuronal time scales embedded in EEG/MEG/fMRI source signals, and to associate changes of dynamical characteristics with brain regions’ functions, physiological states, and conditions^32,34,35,87,88^. In parallel to this important line of research within the reductionist framework, investigations have focused on integrative coupling, coherence, and synchronization phenomena in brain dynamics and network-based approaches to understand emerging behaviors through structural and functional connectivity^5,13,32,37,39–45,47–53,55–57,66,89–97^. Our study focuses on coupling forms and network interactions among dominant and non-dominant brain rhythms that are simultaneously present at a given cortical location during a given physiological state. In addition to understanding how auto-correlation dynamics over various neuronal intrinsic time scales (cortical rhythms) respond to external inputs and change of states, our findings demonstrate the utility of cross-communication and network integration among all cortical rhythms in maintaining brain functions under diverse physiological states. In the broader context of consciousness, cognition, and mind^51,90,95,98^, our findings show that in addition to dynamical patterns embedded in individual cortical rhythms and source signals, dynamic networks of interaction among all cortical rhythms with specific hierarchical structure play an essential role in generating and maintaining cognitive function, and thus, may serve as a mediating substrate (underlying currency^96^) connecting neuronal level signaling with high-level cognitive functions and consciousness.

Within the framework of Network Physiology^99^ where emerging behaviors at the organism level are studied through the prism of interactions among diverse physiological and organ systems^100,101^, our findings open new horizons to investigate network interactions among all cortical rhythms^102–104^, how brain-rhythm cross-communications regulate individual systems^105–107^, and to establish basic principles of multi-component coordination and integration among dynamical systems to generate emerging functions^108–111^, where dynamic maps of brain-rhythm network interactions are utilized as novel diagnostic and prognostic biomarkers in health and disease.

## Methods

### Subjects

Twenty young males, age 19–32 years (average 23.8 years), were recruited from a pool of undergraduate students from the University of Granada, Spain. Participants met inclusion criteria of: reporting >3 h of moderate physical activity per week; normal or corrected to normal vision; no neurological, cardiovascular, or musculoskeletal disorders; no medication in-take. Participants’ fitness level was confirmed by an incremental effort test (see sub-section Fitness assessment). No invasive procedures were involved in the experimental protocol. All subjects gave written informed consent before the study. The protocol was approved following the University of Granada’s ethical guidelines and the Declaration of Helsinki of 1964. One of the participants was subsequently excluded from the final analyses because he did not attend all three protocol tests (fitness assessment and two tests with different levels of physical effort; see sub-sections below). De-identified data from the remaining 19 participants were included in the analyses and reported in this study.

### Fitness assessment

We adopt the ventilatory anaerobic threshold (VAT) as a reference to determine the fitness level of participants. Defined as the volume of Oxygen (VO_2_) when respiratory exchange ratio exceeds one (RER = CO_2_production/O_2_intake)^112,113^, VAT is a sensitive measure of aerobic fitness and cardio-respiratory endurance performance^114,115^. Before the fitness test, descriptive anthropometric parameters (height, weight, and body mass index) were obtained for each participant. Participants then performed an incremental effort cycle-ergometer test to obtain their VATs, which were used to set the individual exercise intensity for each subject in the experimental protocol. We used a ViaSprint 150P cycle ergometer (Ergoline GmbH, Germany) to induce physical effort, obtain pedaling power values, and a JAEGER Master Screen gas analyzer (CareFusion GmbH, Germany) to measure gas exchange during the test. A brief warm-up was introduced for each participant to set a preferred cadence (between 60–90 rev/min), and the subject was asked to maintain it throughout the fitness assessment. The incremental effort test to assess the individual fitness level of each subject started at 60 W pedaling power (minimal resistance) which was kept for 2 min at a steady workload of 60–90 rev/min cycling cadence, followed by 1 min period when the pedaling power increased by 30 W at a rate of 5 W per 10 sec, while keeping the cycling cadence at 60–90 rev/min. Starting at the higher pedaling power (resistance level), the process repeats in ‘2 min + 1 min’ cycles by incrementally increasing the effort with 3 W at the end of each cycle, while keeping the cycling cadence at 60–90 rev/min until the max resistance (exhaustion) was reached. The oxygen uptake (VO_2_, ml/kg/min), RER, relative power output (W/kg), and heart rate (bpm) were continuously recorded throughout the incremental effort fitness assessment test. A participant is considered to have reached his VAT when the RER was maintained above 1.0 during the 2 min constant load period or never reaching 1.1 during the 1 min ramping-up period. Once the VAT was obtained, the corresponding max pedaling power was recorded and used to set the workload (80% or 20% VAT, measured in W) during the exercise sessions in the actual experimental protocol, where gas exchange, oxygen volume VO_2_, and CO_2_ production were not monitored. We note that this individual fitness assessment of each subject is a sub-maximal cardio-respiratory VAT test, since it ends prior to full exhaustion. The fitness assessment was performed for each subject 2–3 days before the actual experimental protocol.

### Experimental protocol

Upon their arrival at the laboratory, all participants received verbal and written information regarding the experimental protocol^59^. Each participant performed two similar experimental protocol tests of 120 min each, at two different physical effort levels—Test-1 at 80% VAT and Test-2 at 20% VAT, while keeping the cycling cadence at 60–90 rev/min. The two tests were separated by at least 48 h and no more than 72 h to avoid possible fatigue or training effects. Subjects were required to maintain a regular sleep-wake cycle for at least 24 h before each experimental protocol test and to abstain from stimulating beverages or any intense physical activity. Each participant attended both tests at the same time of the day to avoid circadian effects on the level of test performance.

#### Sessions

Subjects sat quietly in a comfortable chair in a dimly illuminated, sound-attenuated room with a Faraday cage where they were prepared for electrophysiological measurements. Initial baseline EEG activity was recorded for a 15 min resting period with eyes closed. Participants then started a 10 min warm-up session on the ergometer at a pedaling power (resistance) corresponding to 20% VAT determined in the incremental effort cycle-ergometer test individually for each subject (see Fitness assessment). Following the 10 min warm-up session, participants completed a 30 min cycling session performed at 80% VAT or 20% VAT, measured in W as max pedaling power for each subject. Session power load was counterbalanced across participants, where by random selection half of the subjects performed first the protocol session with 20% VAT effort during exercise followed by the session with 80% VAT effort, and the remaining half performed first the 80% VAT session followed by the 20% VAT session. After an exercise session, a 10 min cooling down period at 20% VAT was completed. Participants were asked to maintain a pedaling cadence between 60–90 rpm throughout the warm-up, exercise, and cool-down sessions, and were instructed to avoid body movements as much as possible and to keep their gaze on a cross at the center of the cycle-ergometer screen. Note that resistance on the cycle-ergometer pedals (during warm-up, exercise, and cool-down sessions) was different for each participant, depending on the individual’s fitness level determined by the incremental effort test, thus allowing for proper comparison among subjects. Upon completing the cooling down session, participants dismounted the ergometer and waited for their heart rate to return to within 130% of their resting heart rate (average waiting time 5.8 min). After that, participants performed a 6 min session of computerized cognitive task, followed by another 15 min resting session with eyes closed, followed by 6 min session of the same cognitive task, which concluded the experimental protocol (see schematic diagram of the entire experimental protocol in Fig. 1a).

#### Cognitive task

We applied a modified version of the Eriksen flanker task^116^ to measure executive control after exercise with different levels of physical effort (see Fitness assessment). A 21-inch monitor and a PC equipped with E-Prime software (Psychology Software Tools, Pittsburgh, PA) were used to present visual stimuli and collect responses from the keyboard. The PC screen center was situated at eyes level, 50 cm from the participant’s head. The cognitive task consists of two types of trials: (i) For congruent trials, a target arrow (at the screen center) is flanked by two arrows on each side pointing at the same direction (e.g., < < < < < or > > > > > ); (ii) For incongruent trials, the target arrow at the center is flanked by two arrows on each side facing the opposite direction (e.g., < < > < < or > > < > > ). Participants were instructed to press the left or right tab button, with the left or right index finger respectively, when the target arrow regardless of trial condition (congruent or incongruent) is pointing to the left (<) or the right (>). Two blocks of 60 trials (randomized across trial conditions) were presented. Each block contains 30 congruent and 30 incongruent trials. Each trial started with the presentation of a white fixation cross on a black background with random duration between 1 s and 1.5 s, followed by a stimulus with duration of 150 ms per trial; subjects response time to stimulus ranged between 400–500 ms, and a variable inter-stimulus interval of 1–1.5 s was introduced at the end of each trial. Each of the two cognitive task sessions approximately lasts for 6 min without a break.

### EEG recording and pre-processing

Continuous EEG data were acquired using a 30-channel actiCHamp System (Brain Products GmbH, Munich, Germany), with active electrodes positioned according to the 10–20 EEG International System, referenced to the Cz electrode and a sampling frequency of 1000 Hz. The EEG cap was adapted to the individuals’ head size, and each electrode was filled with Signa Electro-Gel (Parker Laboratories, Fairfield, NJ) to optimize signal transduction. Electrode impedances were kept below 10 kΩ. Continuous EEG data from six cortical locations (Frontal: Fp1 and Fp2; Central: C3 and C4; Occipital: O1 and O2) were included in the analysis and were pre-processed through (i) a high-pass filter at 0.1 Hz, and (ii) a surface Laplacian spatial filter^117^, where each electrode was re-referenced to the average of neighboring electrodes, providing an approximation of radial current density at each electrode site. The surface Laplacian filter has been demonstrated as a reliable way to distinguish spatial differences in EEG activity and to avoid spurious local coherence due to volume conduction^81^.

Special care was taken to obtain clean EEG recordings during the experimental protocol by controlling external environmental inputs and maintaining steady conditions during each session (physiological state) of the two tests—e.g., controlled sound, light, temperature, and sitting conditions during rest; maintaining constant cycling cadence at 60–90 rev/min during exercise while keeping gaze on a cross at the center of a screen placed in front of the cycle-ergometer; steady stream of cognitive task inputs (visual stimuli) with fixed duration. Each EEG recording was subject to visual inspection and no patches of continuous noise (usually due to poorly attached EEG electrodes) were identified.

### Spectral decomposition

To probe effective cross-frequency interactions among brain rhythms, pre-processed EEG data from 30 channels are segmented into moving windows of 2 s with 1s  overlaps across all seven sessions in the experimental protocol. Within each time window, power spectra of EEG signals are computed using fast Fourier transform and the power *S*(Δ*f*_*i*_) for six physiologically relevant frequency bands is calculated: *δ* [0.5–3.5] Hz, *θ* [4–7.5] Hz, *α* [8–11.5] Hz, *σ* [12–15.5] Hz, *β* [16–19.5] Hz, and *γ* [20-24.5] Hz. The spectral power *S*(Δ*f*_*i*_) in a frequency band Δ*f*_*i*_ is a time series with 1 s resolution representing the dynamics of each physiologically relevant brain-rhythm. To quantify the relative contribution of each brain-rhythm to the total EEG activity, we normalize the band power by the sum of the power in all frequency bands, $\widetilde{S}({{\Delta }}{f}_{i})=S({{\Delta }}{f}_{i})/\mathop{\sum }\nolimits_{i = 1}^{6}S({{\Delta }}{f}_{i})$. A moving average with a 14s  window and a 1 s step is then applied to the time series $\widetilde{S}({{\Delta }}{f}_{i})$. The obtained relative spectral power $\widetilde{S}({{\Delta }}{f}_{i})$ captures not only the quasi-stationary behavior of distinct brain waves during a specific physiological state but also reflects the micro-architecture (1 s resolution) of synchronous modulation in the amplitude of brain waves that gives rise to effective couplings, and allows to track variations in coupling among brain waves with transitions across physiological states (Fig. 1).

### Cross-correlations between brain rhythms

Dynamical coupling of brain rhythms are embedded in continuous and coordinated fluctuations of EEG band powers. To uncover cross-communications and couplings between distinct brain rhythms that occur as a result of synchronous modulation of their spectral amplitudes at short time scales of a few seconds, we divide the relative spectral power time series $\widetilde{S}({{\Delta }}{f}_{i})$ of each brain-rhythm into *N*_*L*_ equally sized, non-overlapping segments of length $L=30\ \sec$ (${N}_{L}=\left\lfloor N/L\right\rfloor$, where *N* is the length of the time series). In each segment, the relative spectral power is converted into *z*-score, $\widetilde{S}({{\Delta }}{f}_{i})\to s({{\Delta }}{f}_{i})\equiv [\widetilde{S}({{\Delta }}{f}_{i})-\langle \widetilde{S}({{\Delta }}{f}_{i})\rangle ]/{{{{{{{\rm{std}}}}}}}}(\widetilde{S}({{\Delta }}{f}_{i}))$ with respect to the mean $\langle \widetilde{S}\rangle$ and standard deviation ${{{{{{{\rm{std}}}}}}}}(\widetilde{S})$ evaluated within the segment. The cross-correlation matrix *C* is then defined as

1 $${C}_{6\times 6}=\frac{1}{L}{\left[\begin{array}{l}-{s}_{\delta }-\\ -{s}_{\theta }-\\ -\cdots -\\ -{s}_{\gamma }-\end{array}\right]}_{6\times L}{\left[\begin{array}{llll}| &| &| &| \\ {s}_{\delta }^{T}&{s}_{\theta }^{T}&\cdots &{s}_{\gamma }^{T}\\ | &| &| &| \end{array}\right]}_{L\times 6},$$

where each matrix element *C*_*ij*_ is a normalized inner-product between two spectral power vectors *s*(Δ*f*_*i*_) and *s*(Δ*f*_*j*_),

2 $${C}_{ij}=\frac{1}{L}\left\langle s({{\Delta }}{f}_{i}),s({{\Delta }}{f}_{j})\right\rangle =\frac{1}{L}\mathop{\sum }\limits_{t=1}^{L}{s}_{t}({{\Delta }}{f}_{i})\ {s}_{t}({{\Delta }}{f}_{j}).$$

The pairwise cross-correlation *C*_*ij*_ takes values between −1 (fully anti-correlation) and +1 (fully positive correlation), with *C*_*ij*_ = 0 indicating the absence of linear relation between two frequency bands Δ*f*_*i*_ and Δ*f*_*j*_ in a 30 s window (Fig. 1c).

The proposed method is robust to effects of noise present in EEG recordings. High-frequency noise (isolated spikes) is removed in the process of band pass filtering—note that the frequency bands of the selected six cortical rhythms are in the lower frequency range (time series of spectral power in Fig. 1a-b). Further, special consideration was given to potential common shocks to EEG signals from the environment (e.g., electric grid) that transcend all frequency bands. Indeed, such shocks would affect the absolute power of each spectral band, leading to spurious effects of cross-correlation (coupling) between cortical rhythms due to global trends of increase or decrease in the spectral power of all bands. We consider the relative contribution of each frequency band to the total EEG spectral power to avoid such effects (Supplementary Fig. 8). Specifically, we perform tests to validate our analyses based on the relative spectral power, and we find spurious positive correlation profiles with no differentiation among different pairs of cortical rhythms within a given physiological state when the absolute spectral power is considered in each frequency band (Supplementary Fig. 8). This is in contrast to the uncovered three distinct classes of cortical rhythm coupling forms and the pronounced stratification in coupling strength with transitions across states (Fig. 2). This demonstrates that considering the relative spectral power is essential to quantify the micro-architecture of synchronized, short-time modulations in the amplitude of brain rhythms that occur on top of their quasi-steady-state behavior and large timescale trends, and give rise to different forms of coupling profiles.

Further, random isolated noisy spikes in EEG signals from the environment follow certain i.i.d. processes with Gaussian amplitudes and Poisson inter-event distribution, are homogeneously dispersed in time and are present in the entire frequency domain (across all EEG bands/brain waves). By taking (i) the relative spectral power of each cortical rhythms over consecutive, non-overlapping windows, and (ii) cross-correlating spectral amplitudes of cortical rhythms over large (30 s) windows, the effects of random isolated spikes are canceled out. The advantage of the proposed novel method (Synchronous Amplitude Network Analysis, SANA) is that it does not require excessive signal pre-processing, and can be adapted to different scales of analysis.

We note that phase synchronization and phase-amplitude coupling measures are based on the instantaneous phase and amplitude of a given cortical rhythm, or across rhythms, extracted from the original signals or band-passed in the frequency range, and thus, probe the dynamics at very short time scales comparable to the sampling rate^32,36–38,41,47,48,50,52,53,56,66,93^. Moreover, the canonical formalism of synchrony is based on the assumption of weakly coupled self-sustained oscillators with small frequency mismatch^118,119^. Our empirical observation that coordinated modulation in the spectral power of cortical rhythms with different frequencies during homeostatically maintained physiological states occurs at larger time scales (20–30 s) over quasi-steady cortical activity implies strong interactions among all cortical rhythms. The proposed SANA method would effectively quantify distinct forms of coupling for cortical rhythm interactions over various time scales, and infer the embedded network structure based on coupling strength.

### Cross-correlation distribution profiles and degree of coupling between brain rhythms

During a specific physiological state (protocol session), synchronous modulation in spectral power amplitudes among brain rhythms is quantified by the distribution of cross-correlation values {*C*_*ij*_} for each pair of brain rhythms obtained from consecutive, non-overlapping 30 s windows in the spectral power time series, derived from the EEG signal at a given channel location. For each pair of brain rhythms, a cross-correlation distribution profile is obtained for individual subjects in each physiological state; {*C*_*ij*_} data from 30 s windows pooled from all subjects are used to obtain group-averaged distribution profiles and to derive degree of coupling between rhythms. A histogram of cross-correlation matrix elements *C*_*ij*_ is obtained by dividing the range [−1, 1] of cross-correlation values into bins of size Δ*c* = 0.05. The histogram is then rescaled by the max number of counts and smoothed with a 5-bin moving average that outlines the distribution profile for each pair of brain rhythms at each physiological state (Figs. 1c and 2, Supplementary Fig. 2). The obtained distribution profile is proportional to the probability density of cross-frequency coupling between brain rhythms that occurs at short-time scales.

Since in our analysis, the relative spectral power time series $\widetilde{S}({{\Delta }}{f}_{i})$ of cortical rhythms are divided into 30 s non-overlapping windows, where cross-correlation values {*C*_*ij*_} between all pairs of rhythms are obtained, there are certain limitations imposed by the length of data recordings during each physiological state in the experimental protocol on the total number data points used to generate the cross-correlation distribution profile for each pairs of cortical rhythms at a given state. For the group average data of the cross-correlation distribution profiles (shown in Fig. 2), the number of *C*_*ij*_ values used to generate each profile ranges from 556 points (for the short 6 min cognitive task segment) to 2280 points (for the 30 min exercise) when pooling together the corresponding sessions (rest, exercise, and cognitive task) from the two separate experimental tests (with 80% VAT and 20% VAT physical effort). These statistics are sufficient to differentiate profiles (coupling forms) for all pairs of cortical rhythms at a given state, and allow for differentiation among profiles and coupling strengths for each pair of rhythms across states, even when we consider the two experimental tests (80% VAT and 20% VAT physical effort) separately (Supplementary Fig. 2).

We note that the morphology of EEG dynamics in the context of particular physiological states or cognitive tasks imposes certain limitations on the minimal length of data recording when our method can be reliably applied. Multiple time scales are embedded in the EEG signals as a result of inputs integration from neuronal units and neuronal assemblies with different temporal dynamics across brain areas. Consequently, different EEG morphology and micro-architecture characteristics can be probed at different time scales. For steady conditions where a given physiological state is homeostatically maintained (as is the case for the resting state, steady exercise, and cognitive task condition in our experimental design), we observe synchronous/asynchronous modulation in the spectral power of cortical rhythms at time scales of around 30 s. Thus, our approach focuses on cross-communication among cortical rhythms at this and larger time scales, which also imposes limitations on the minimal length of EEG recordings necessary to identify and quantify cortical rhythm coupling profiles (Fig. 2 and Supplementary Fig. 2), coupling strengths, and network interactions (Fig. 3 and Supplementary Figs. 3 and 4). Our analyses and statistical tests show that continuous, high-frequency (1000 Hz) EEG recordings of 5–10 min length is required. This falls within the majority of basic physiological and clinical test protocols, and thus, allows for application of the proposed method to broad range of states/conditions. In the case of studying instantaneous responses to short-term perturbations, e.g., Event Response Potentials (ERPs), the proposed Synchronous Amplitude Network Analysis (SANA) method can be flexibly adapted to modulation patterns at much shorter time scales, and thus, would require smaller windows of a few seconds and shorter continuous EEG recordings to quantify instantaneous responses to perturbations in cortical rhythm coupling and network structure.

Based on results of statistical tests from randomized data (see Statistical tests), we find a threshold ∣*C*_0_∣ = 0.5 that distinguishes physiologically significant positive cross-correlation (*C*_*ij*_ > 0.5) and significant anti-correlation (*C*_*ij*_ < −0.5) between two rhythms from accidental correlations in random data. We introduce two symmetric matrices *D*^±^ to characterize the degrees of cross-correlation among brain rhythms (EEG frequency bands). The matrix elements ${D}_{ij}^{\pm }$ represent the probability of observing significant positive cross-correlation, ${D}_{ij}^{+}=P({C}_{ij} \, > \,0.5)$, and the probability of observing significant anti-correlation, ${D}_{ij}^{-}=P({C}_{ij} < -\!0.5)$, respectively.

Each physiological state is represented by two matrices *D*^±^, where matrix elements ${D}_{ij}^{\pm }$ are the probabilities of finding strongly coupled (positively or negatively cross-correlated) pairs of brain rhythms. Empirically, the matrix elements ${D}_{ij}^{\pm }$ are a measure of the fraction of time when statistically significant cross-correlation values are observed during a protocol session (distinct physiological state). Numerically, ${D}_{ij}^{\pm }$ are estimated as areas under the distribution profile that are above or below the significant threshold *C*_0_ = ±0.5, normalized by the total area under the profile (see schematic diagram in Supplementary Fig. 3).

The degree of coupling between brain rhythms (i.e., *D*^±^) for a representative subject and for the entire group is shown as bar plots in Fig. 3 with positive bars for *D*^+^ and negative bars for *D*^−^. The matrices *D*^±^ are calculated for all physiological states (protocol sessions) and all EEG channel locations representing different brain areas in the left and the right hemispheres (Supplementary Figs. 13–15).

### Networks of brain-rhythm interactions

The degree of cross-correlations is a concise characterization of brain-rhythm interactions. It reduces the distribution of cross-correlation values {*C*_*ij*_}, obtained for multiple 30 s windows from spectral power time series, to two numbers (probabilities ${D}_{ij}^{\pm }$) while preserving essential features (e.g., skewness) of the distribution. To dissect the complex cross-frequency coupling, we construct two sets of dynamic networks based on *D*^±^ for positive correlations and anti-correlations among the six brain rhythms (Figs. 4b and 5b). Network links correspond to the group-averaged degree of cross-correlation coupling for different pairs of brain rhythms derived from our Synchronous Amplitude Network Analysis (SANA) method (links are linearly scaled with thickness and color; thicker and darker lines represent stronger coupling). The coexistence of both positively and anti-correlated networks for each physiologic state demonstrates a complex duality and a transient nature of brain-wave communication. Coupling between brain rhythms can switch on or off at different times during the same physiologic state. The emergence of distinct sub-clusters in both networks and their reorganization with transitions across physiological states indicate that network communications among brain rhythms play a key role in physiologic regulation.

We note that in the introduced here SANA method, the coupling measure between brain rhythms represents amplitude–amplitude coupling since we cross-correlate the relative spectral power of brain rhythms in non-overlapping windows over the time course of the EEG recording for each session (physiological state) in the experimental protocol. Because we have non-overlapping 30 s windows in which amplitude–amplitude cross-correlation values are obtained, this measure reflects synchronous amplitude modulation of cortical rhythms over their time course at a given location. Our analyses show that different pairs of brain rhythms cross-communicate through the different degrees of synchronous/asynchronous modulation at a given state, and that the spectral amplitude modulation between cortical rhythms changes for different physiological states leading to different network configurations of coupling strengths among cortical rhythms.

### Statistical tests

#### Control for different time scales of analyses

The aim of our study is to quantify synchronous amplitude modulation in the spectral power of cortical rhythms within the quasi-steady-state behavior of the total EEG power during a given physiological state. Our approach is motivated by cortical rhythm dynamics showing bursting activity in brain-waves ranging over seconds to minutes. The choice of 30 s cross-correlation time window in our analysis is guided by modulation patterns in the relative spectral power of cortical rhythms with quasi-periodicity of two to five waves within segments of 100 s (red lines in Fig. 1a, b and Supplementary Fig. 1). This is also aligned with American Association of Sleep Medicine guidelines for accessing physiological states (sleep/wake, sleep stages, quiet rest) where 30 s are used as a basic epoch. Thus, within large time scales of 5–30 min during a given state, homeostatically maintained at steady conditions as in our experimental protocol, our analyses focus on the micro-architecture embedded in the quasi-stationary dynamics of cortical rhythms.

To examine the effect of different sizes for the cross-correlation time window, we repeated our analyses over a range of time scales, yielding consistent results for the ensemble of coupling profiles and coupling strengths among cortical rhythms for all physiological states (Supplementary Fig. 5).

Further, we tested the effect of the moving average window size while keeping the cross-correlation window fixed at 30 s. We note that a too-short window of just a few data points does not have the desired smoothing effect to reveal synchronous modulation in the spectral power time series of different brain rhythms at the 1s resolution of our spectral power analysis. On the other hand, a smoothing window that is too long (longer than half the chosen cross-correlation window) will carry information from neighboring data segments, and thus, influences the coupling information derived from our analysis within each cross-correlation window. Repeating the analyses over a range of moving average windows on the relative spectral power time series for fixed 30 s cross-correlation windows, we confirm the validity of our findings of distinct coupling profiles between cortical rhythms, their organization in three separate classes, and modulation in coupling profiles and coupling strengths depending on physiological states (Supplementary Fig. 6). The performed additional tests confirm that our findings are robust over a range of time scales.

#### Collapse of individual profiles on group averages

To test the consistency of pairwise coupling profiles across subjects (Supplementary Fig. 7), we ran Wilcoxon signed-rank tests on the distribution profile of each pair of brain rhythms from an individual subject and the corresponding accumulated group average profile for each physiological state (the subject being excluded from the group average calculation). The null hypothesis (*h* = 0) is that there is no difference between the individual distribution and the group average distribution profile. Results show that a high percentage of individual profiles (>87%) passed the tests (*p* > 0.05)—i.e., the majority of subjects share the same distribution form as the group profile. Statistically, no more than three subjects (out of 19) have cross-correlation distribution profiles that differ (with 95% confidence level) from the group averaged coupling profile for all pairs of brain rhythms and all physiological states (protocol sessions).

#### Correlation distributions from absolute spectral power

Since the EEG amplitude heavily influences oscillations across all frequency bands (i.e., brain rhythms at the specific EEG channel location), applying the analysis to the absolute spectral power of each frequency band results in strong positive correlations within the 30 s windows for all pairs of brain rhythms, lacking differentiation of brain-rhythm interactions as a function of physiological state (Supplementary Fig. 8). The reason is that large-scale trends and modulation in EEG amplitude (due to intrinsic physiologic regulation or external factors such as movement artifacts and changes in scalp connectivity) lead to global change in the whole power spectrum and positive cross-correlations among all frequency bands, and thus, mask short-time synchronization in the spectral power of cortical rhythms that mediate their interactions. Considering the relative spectral power of brain rhythms reduces these confounding factors, preserving each frequency band’s relative contribution to the total spectral power and reveals physiologically relevant coupling profiles that vary among brain rhythms for distinct physiological states (Fig. 2 and Supplementary Fig. 2).

#### Surrogate test on shuffled data

To test the robustness of our results and to determine the significance threshold (∣*C*_0_∣ = 0.5) in the degree of coupling for all pairs of brain rhythms, we perform surrogate analysis where normalized and smoothed spectral power time series of the brain rhythms are shuffled before calculating cross-correlation in 30 s windows (Supplementary Fig. 9). Gaussian-shaped distribution profiles of cross-correlation from the surrogate test for all pairs of brain rhythms and all physiological states are centered at *C* = 0 and decay to zero near *C* = ±0.5 (93% of *C*_*ij*_ values fall in the interval between −0.5 and +0.5). This shuffled data surrogate test shows that the degree of positive cross-correlation (*C* > 0.5) and anti-correlation (*C* < −0.5) does not result from a random process but represents physiologically relevant coupling. Thus, the measure we introduce for the degree of positive and anti-correlated coupling—normalized areas under the coupling profile for each pair of brain rhythms that are beyond the threshold ∣*C*_0_∣ = 0.5 (i.e., matrix elements ${D}_{ij}^{\pm }$, depicted as bars in Fig. 3 and Supplementary Figs. 3 and 4, see Cross-correlation distribution profiles and degree of coupling between brain rhythms in Methods)—represents real physiological interactions among brain rhythms. We find that the degree of coupling for each pair of brain rhythms does not significantly change when varying the threshold |*C*_0_ | = {0.3, 0.4, 0.6, 0.7}, indicating robustness of our finding of three major classes of brain-wave interactions and pronounced stratification across distinct physiological states of rest, exercise and cognitive task (Fig. 3).

#### Cross-correlation *p*-value distributions

The statistical significance of our results is further confirmed by the distribution of the *p*-values for the Pearson cross-correlation coefficients {*C*_*ij*_} for all 30 s windows from all subjects. *P*-value distributions are centered below *p* = 0.05 (Supplementary Fig. 10a) with >96% of *p*-values below *p* = 0.05, indicating statistical significance of the cross-correlation values {*C*_*ij*_} and coupling profiles observed in the data for all pairs of brain rhythms and all physiological states (protocol sessions), shown in Figs. 2 and 3. In contrast, *p*-values of cross-correlation coefficients in 30 s windows pooled from shuffled spectral power time series of each frequency band exhibit a uniform distribution with >96% of the *p*-values above *p* = 0.05, thus verifying the null hypothesis that the analyzed data are random samples (Supplementary Fig. 10b).

#### Surrogate test on random subjects data

To further validate the physiological origin of the observed brain-rhythm coupling profiles, we analyzed cross-correlation for pairs of brain rhythms where the spectral power time series for each rhythm was taken from a different subject during the same physiological state. Surrogate test coupling profiles for all pairs of brain rhythms were obtained pooling together cross-correlation {*C*_*ij*_} values from 30 s windows in 19 realizations of surrogate pairs, where a brain-rhythm of a subject at a given physiological state was paired with a different brain-rhythm from a different, randomly chosen subject. While actual data exhibit short timescale amplitude modulations on top of quasi-steady states in the spectral power of cortical rhythms (Fig. 1a and b), these amplitude modulations would not synchronize for different subjects, and thus, would not result in consistent brain-wave interaction profiles, leading to uniform distribution of cross-correlation {*C*_*ij*_} values for all pairs of brain rhythms, with no differentiation between pairs (Supplementary Fig. 11a) and no stratification for physiological states (Supplementary Fig. 11b). This surrogate random subjects test confirms that the reported major classes of brain-rhythm coupling profiles (Fig. 2, Supplementary Fig. 2) and stratification in network interactions among brain rhythms across physiological states (Figs. 4 and 5) reveal genuine physiological phenomena.

#### Surrogate test on Fourier phase randomization

To demonstrate the physiological significance of brain-rhythm interactions, we also performed a Fourier phase randomization^120–124^ on EEG signals, which preserves the global spectral power of different frequency bands (brain rhythms) within the recording but destroys phase information that relates to nonlinear EEG characteristics, and thus, eliminates the fine temporal structure in the spectral dynamics and coupling between brain rhythms. In Fourier-phase-randomized surrogate data, the relative ratios among the average spectral power of brain rhythms are preserved, while synchronous modulations in frequency bands that underlie effective cross-frequency coupling and account for the nonlinear characteristics of EEG signals are eliminated. The surrogate test shows a different set of interaction profiles (Supplementary Fig. 12) compared to the actual data (Fig. 2): although the three major classes of coupling between brain waves are still present, the shape of coupling profiles for specific pairs of brain rhythms is modified at each physiological state. Specifically, while the class of anti-correlated pairs of brain waves is still visible, the coupling profiles have modified shapes compared to real data. In contrast, the other two classes (with mixed and positive correlations) exhibit dramatically altered coupling profiles for the surrogate data. These results indicate that the distinct coupling forms reveal physiological information about (i) the relative difference in the total spectral power at large time scales during a physiologic state and (ii) the synchronous modulation of brain-wave spectral amplitudes at short time scales.

### Reporting summary

Further information on research design is available in the Nature Research Reporting Summary linked to this article.

## Supplementary information

Supplementary Materials

Description of Additional Supplementary Files

Supplementary Data 1

Reporting Summary

## Data Availability

The data analyzed in this work are multi-channel EEG recordings from the REXCO Project. All source data underlying graphs are enclosed in the Supplementary Information as an Excel spreadsheet Supplementary Data 1.

## Code availability

MATLAB codes for data processing are available upon request from the corresponding author.
