# Supplementary material for: Ensemble of coupling forms and networks among brain rhythms as function of states and cognition
Source: Commun Biol. 2022 Jan 21;5:82. doi: 10.1038/s42003-022-03017-4 (PMC8782865; doi:10.1038/s42003-022-03017-4)
Supplement: Supplementary file 1 — Supplementary Materials [file 42003_2022_3017_MOESM1_ESM.pdf]

Supplementary Figures

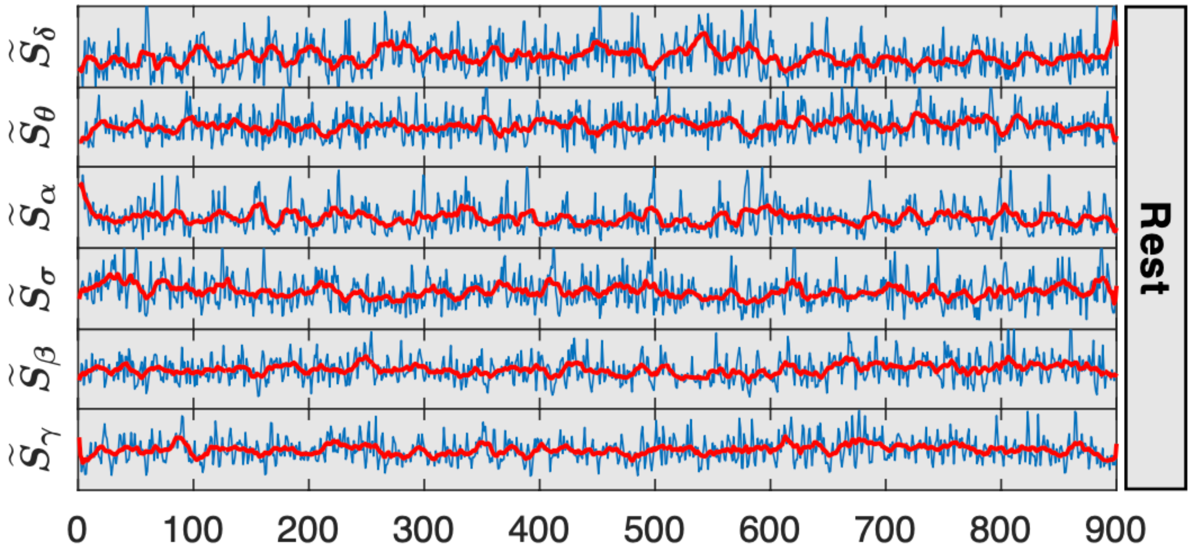

Supplementary Figure 1. Modulation patterns in spectral power micro-architecture of cortical rhythms reflect underlying interactions. Time series of relative spectral power $\tilde{S}(\Delta f_i)$ derived from the EEG C3 channel of a representative subject during a segment of homeostatically maintained resting state (blue lines; x-axis time units in seconds). Moving averages (red lines) show amplitude modulation and coordination among cortical rhythms with on/off periods of synchronous/asynchronous modulation patterns in the relative spectral power time series with durations that span from seconds to minutes, indicating dynamic interactions between *dominant* and *non-dominant* brain rhythms.

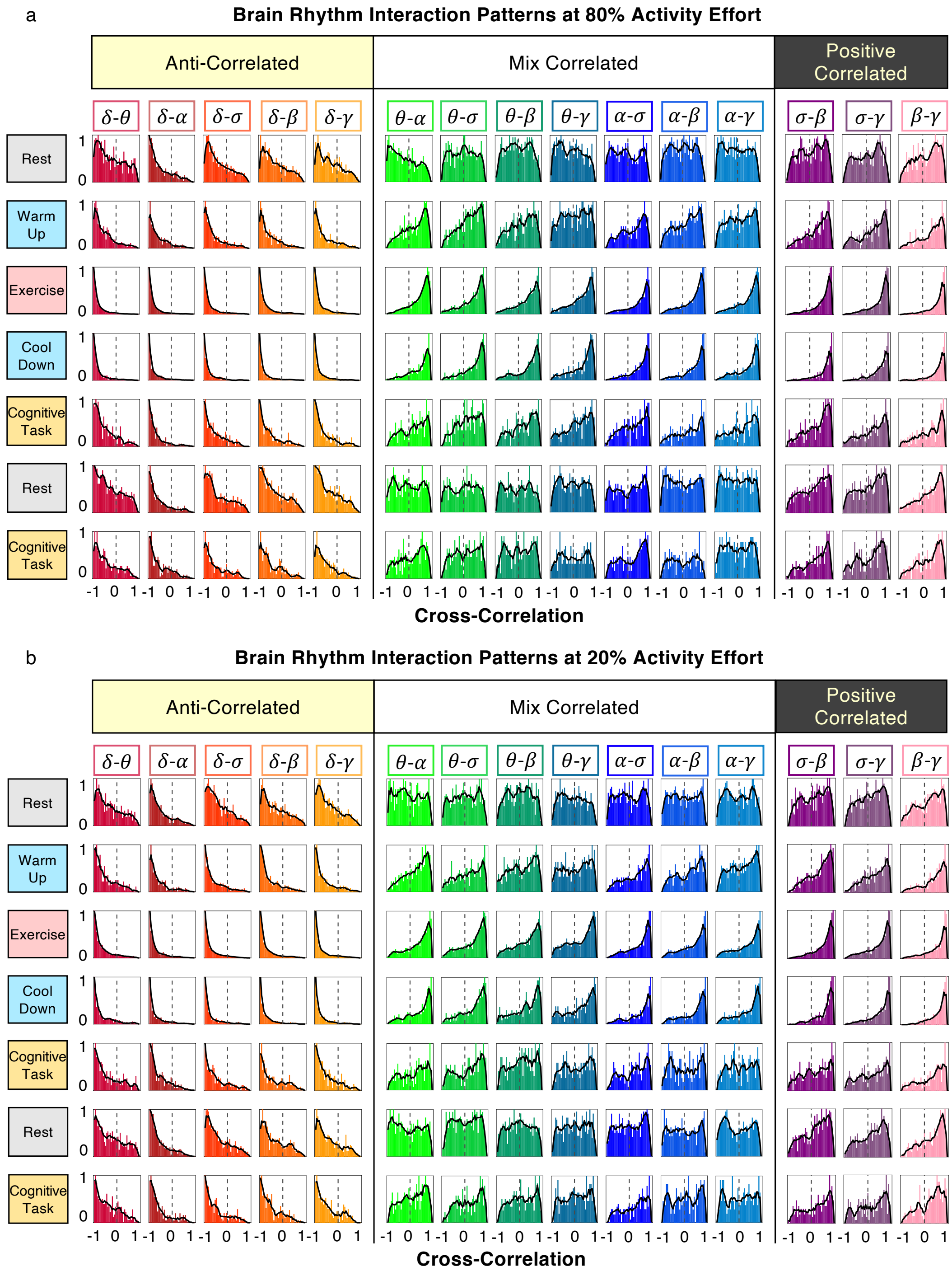

Supplementary Figure 2. Coupling profiles of brain rhythm interactions show consistency for tests performed at different levels of physical activity effort. Cross-correlation distribution profiles for each pair of brain rhythms during distinct physiological states (rest, warm-up, exercise, cool-down, cognitive task), where the same experimental protocol test was repeated on different days for (a) high and (b) low physical effort during exercise with respectively 80% and 20% ventilatory anaerobic threshold (VAT) (see Methods). Coupling profiles for each pair of brain rhythms (derived at the C3 EEG channel location) are obtained by pooling data from all subjects. Solid black lines represent smoothed group-averaged coupling profiles. Three major classes of brain rhythm interactions (with anti-correlated, mix correlated and positively correlated profiles; Figure 2) are consistently observed across all physiological states for both tests. Such robustness to repeated protocol tests at different levels of physical effort demonstrates the presence of a fundamental mechanism regulating interactions among cortical rhythms, and that an ensemble of interaction profiles (an ‘alphabet’ of brain rhythm communications) uniquely defines each physiological state.

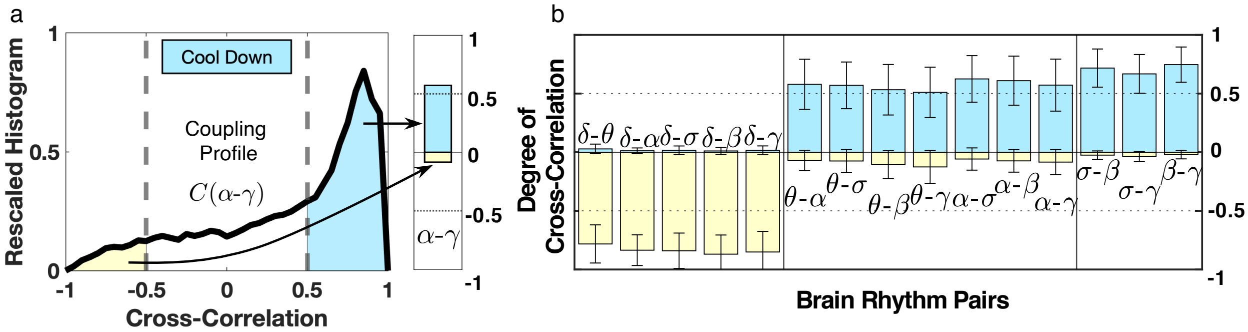

Supplementary Figure 3. Degree of cross-correlation as a measure of brain rhythm interactions. a Schematic diagram representing the degrees of significant positive cross-correlation and negative anti-correlation between α and γ waves during cooling down from exercise (Experimental sessions protocol, Methods), calculated as ratios of areas (blue and yellow, respectively) under the rescaled histogram profile $C(\alpha\text{-}\gamma)$ (solid line), that are beyond the significance threshold $|C| > 0.5$, and the total area under the curve (shown as positive and negative bars). The derived coupling measure is numerically equal to the probability (fraction of total time during a physiological state) of observing significant positive and negative cross-correlations beyond the significance threshold (determined in Supplementary Figure 9). **b** Bar plot shows the degrees of cross-correlation between all pairs of brain rhythms during the physiological state of cooling down from exercise. Positive bars are in blue corresponding to the physiological state and negative bars in yellow. Error bars represent group standard deviation.

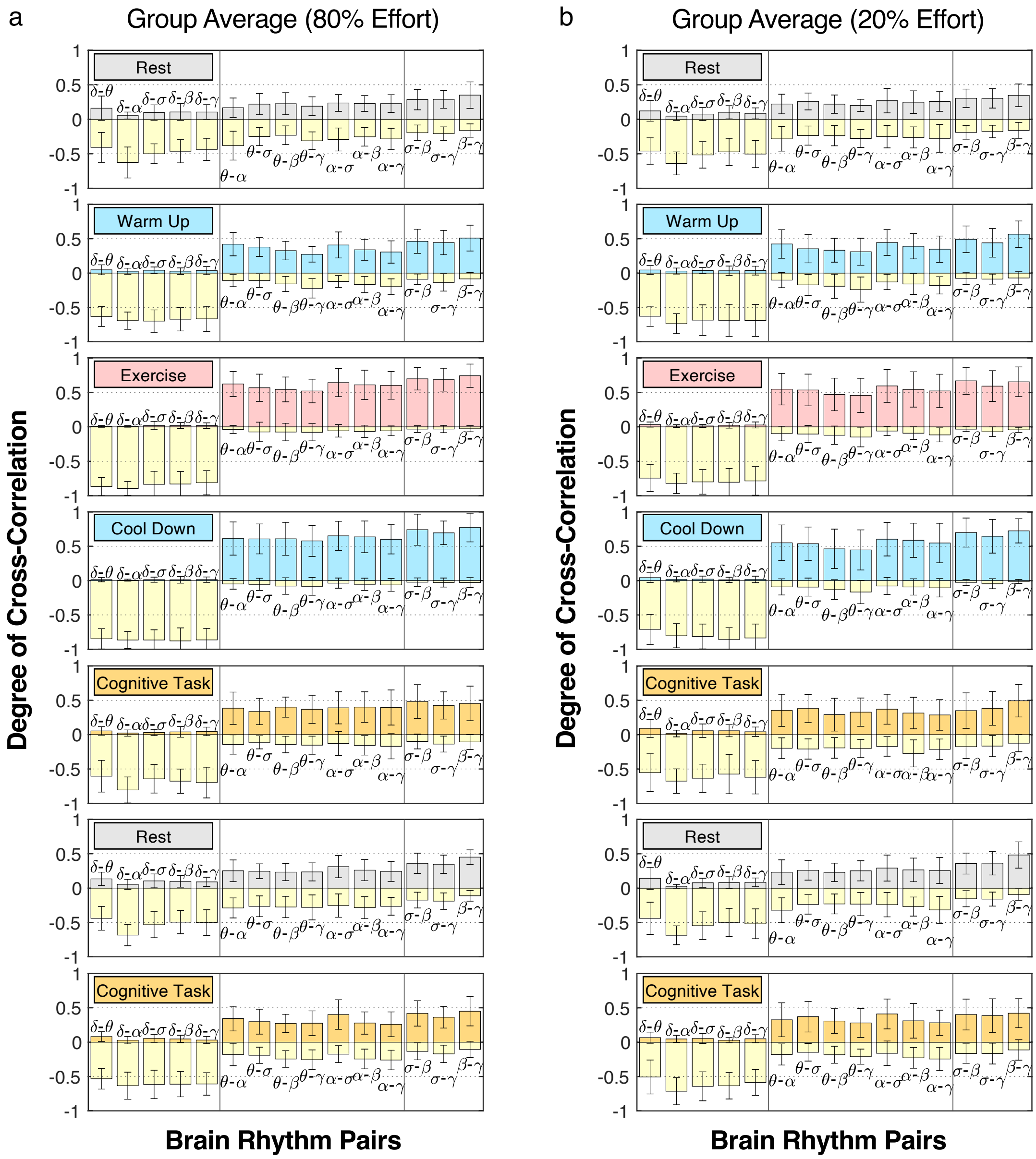

Supplementary Figure 4. Reorganization of interactions among brain rhythms with transitions across physiological states. Robust stratification emerges when comparing the degree of positive and negative coupling between all pairs of brain rhythms (at the C3 EEG channel location) for different physiological states, across subjects, and for repeated protocol tests with (a) high 80% VAT and (b) low 20% VAT physical activity effort during exercise – very high degree of only negative or only positive coupling for all pairs of brain rhythms during exercise and cooling down segments of the protocol; intermediate degree of coupling during repeated segments of the same cognitive task; and lower degree of coupling for resting periods where each pair of brain rhythms exhibits partially both positive and negative coupling. Positive bars are shown in colors corresponding to the physiological states and negative bars in yellow. Error bars represent group standard deviation. Notably, despite reorganization in brain rhythm interactions with transitions across distinct physiological states, three major classes of coupling forms are present, i.e., pairs of rhythms with (i) strong anti-correlated and (ii) strong positively correlate coupling across all states, and (iii) mixed coupling profiles that change across states (Figures 2 and 3). These observations indicate a robust mechanism of synchronous modulation of bursting activity in the micro-architecture dynamics of cortical rhythms, and that a specific of organization of interactions among cortical rhythms is essential to maintain physiological states and generate functions.

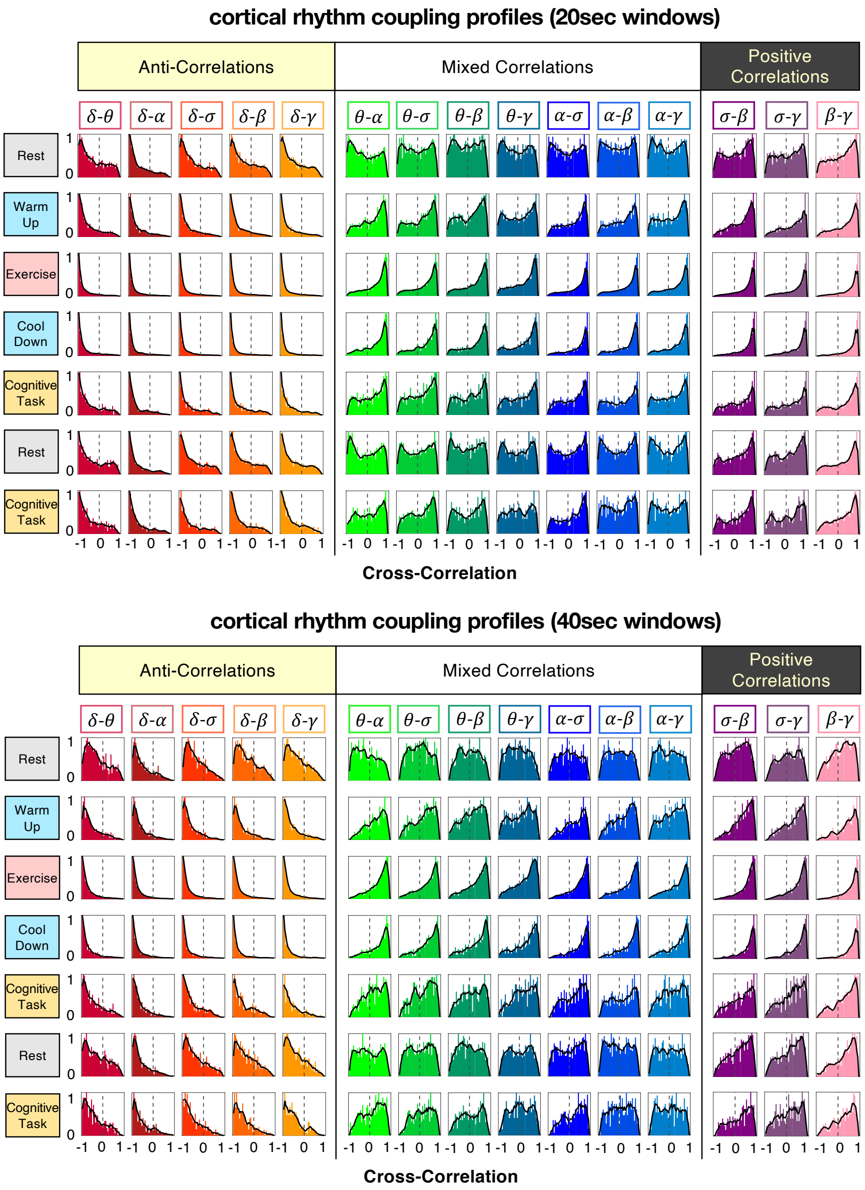

Supplementary Figure 5. Coupling profiles and transitions in brain-rhythm interactions across physiologic states remain robust for different time scales of analysis. Cross-correlation distribution profiles for each pair of brain rhythms obtained for 20s (top panel) and 40s (bottom panel) time windows (after 14s moving average is applied to the relative spectral power time series for each cortical rhythm) during different physiologic states. Data are pooled from all subjects in the database from two separate tests repeating the same protocol on different days at high (80% VAT) and low (20% VAT) level of physical effort during exercise (Methods). Distributions are rescaled by the peak values of cross-correlation and smoothed by moving average (solid black lines). Practically identical results are obtained for both 20s and 40s time windows, indicating that the established coupling profiles are robust and present over a broad range of time scales for our SANA method (Methods). For both time windows and for all physiological states, interactions among distinct brain rhythms fall in three major classes: (i) anti-correlated coupling with predominantly negative correlation values for the pairs δ-θ, δ-α, δ-σ, δ-β, δ-γ; (ii) positively correlated coupling with predominantly positive correlation values for the pairs α-σ, α-β, α-γ, σ-β, σ-γ, β-γ; and (iii) mixed correlated coupling for θ-α, θ-σ, θ-β, θ-γ, where the coupling profile gradually changes (along each column) from weakly negative to homogeneous and positive. Within these classes and for both 20s and 40s time windows (top and bottom panels), coupling profiles for each pair of cortical rhythms exhibits consistent modulation with transitions across states, e.g., fast-decaying tail during exercise (strong coupling), slow-decaying tail during the cognitive task (intermediate coupling), and fat tail during rest (weaker coupling). The same ensemble of profiles (along each row) characterizes a given physiological state for both time windows of analysis. These findings indicate that a specific set of brain-rhythm coupling forms (an 'alphabet' of profiles) is robust over time scales of observation and uniquely defines each physiologic state, and that a complex hierarchical organization in the cross-communication among brain rhythms is a hallmark of physiologic states and functions.

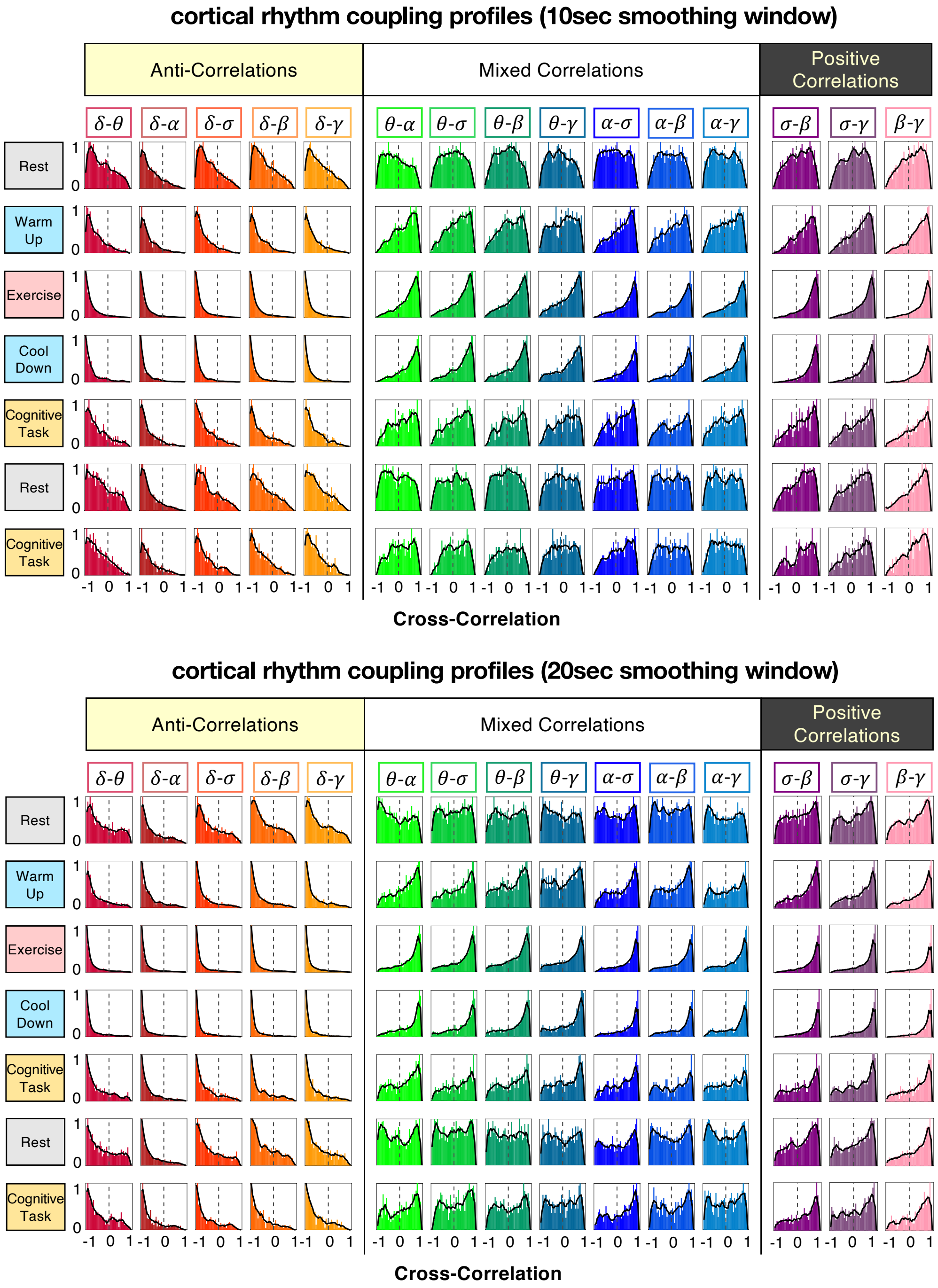

Supplementary Figure 6. Coupling profiles obtained for different moving average window sizes applied to smooth spectral power time series of cortical rhythms. Coupling profiles for each pair of brain rhythms obtained from cross-correlation values in non-overlapping 30s time windows after 10s (top panel) and 20s (bottom panel) moving average is applied to the relative spectral power time series for each cortical rhythm) during different physiologic states. Results are consistent with those obtained for a 14s moving average window (Figure 2), indicating robustness of the coupling profiles results. Data are pooled from all subjects in the database from two separate tests repeating the same protocol on different days at high (80% VAT) and low (20% VAT) level of physical effort during exercise (Methods). Distributions are rescaled by the peak values of cross-correlation and smoothed by moving average (solid black lines).

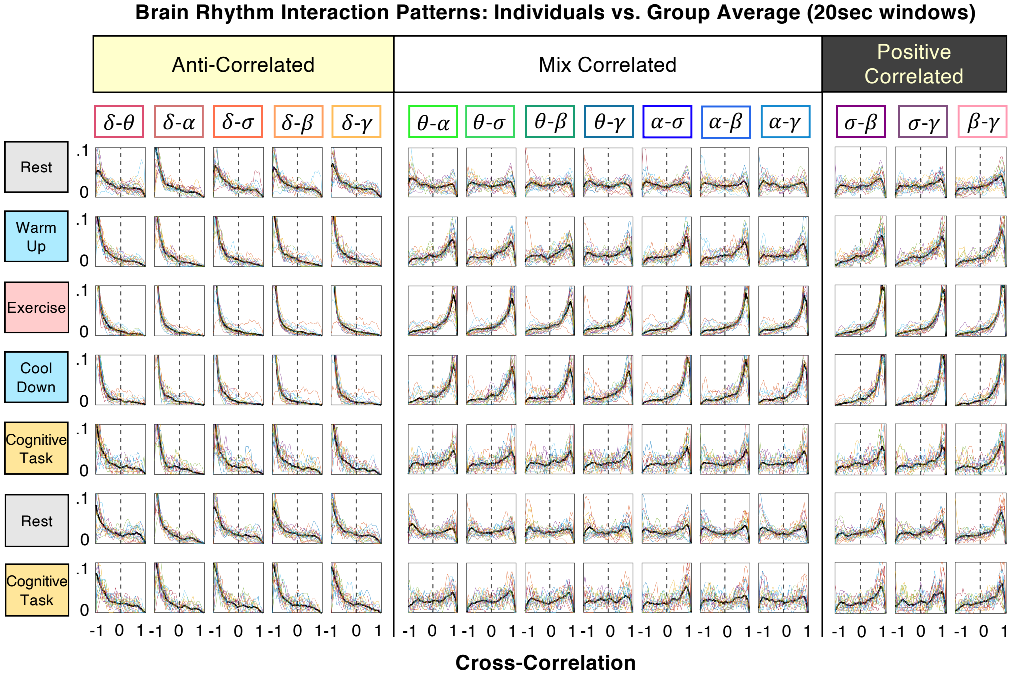

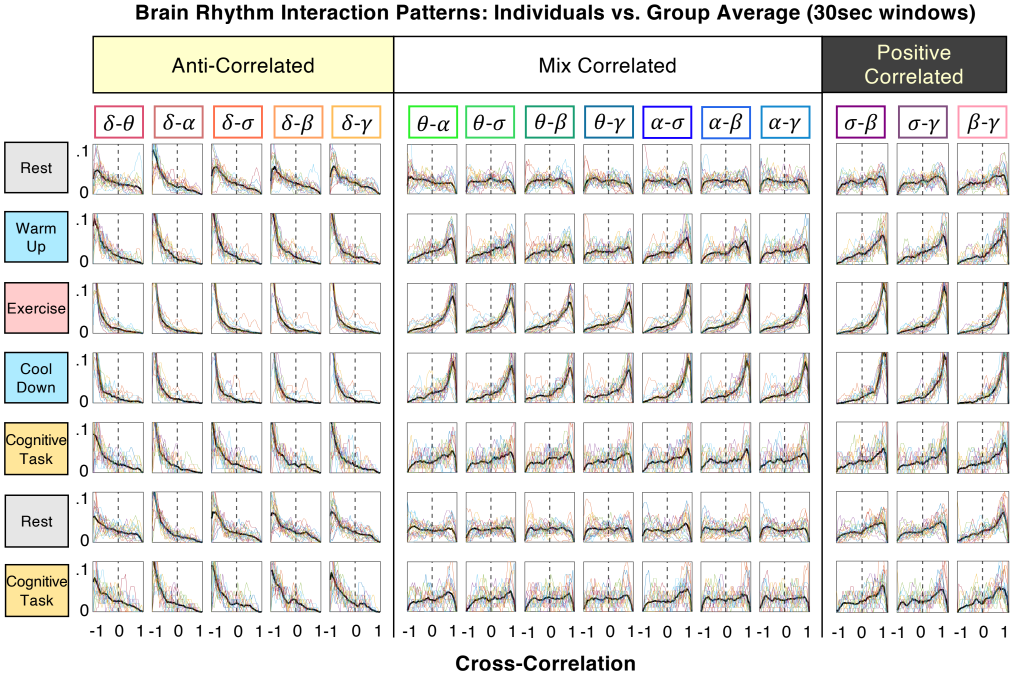

Supplementary Figure 7. Universal patterns of brain wave interactions across subjects and states. Cross-correlation distribution profiles representing coupling forms for all pairs of brain rhythms at the C3 EEG channel location during distinct physiologic states. Cross-correlation values are computed in non-overlapping 30s windows during each physiological state (see Methods). Cross-correlation distribution profiles for each subject are obtained for each pair of brain rhythms at each physiological state by pooling together cross-correlation values from the corresponding sessions in two repeated experimental protocols with different levels of physicial effort – Test-1 at 80% VAT and Test-2 at 20% VAT (see Methods). Curves in color represent the probability distribution of cross-correlation values for individual subjects; black solid lines show the group average obtained by pooling data from all subjects. For each pair of brain waves during a given physiological state (row of panels), all individual subjects' distributions collapse and conform to a single shape (coupling profile) with 95% confidence level (Wilcoxon signed-rank test), indicating that the functional form of coupling for each pair of brain rhythms is universal for all subjects. Remarkably, data collapse of the cross-correlation distribution profiles is consistently observed for all pairs of brain rhythms across all physiological states, indicating the presence of an 'alphabet' of brain wave communications (a set of coupling profiles) that uniquely characterizes each physiological state at the individual subject level.

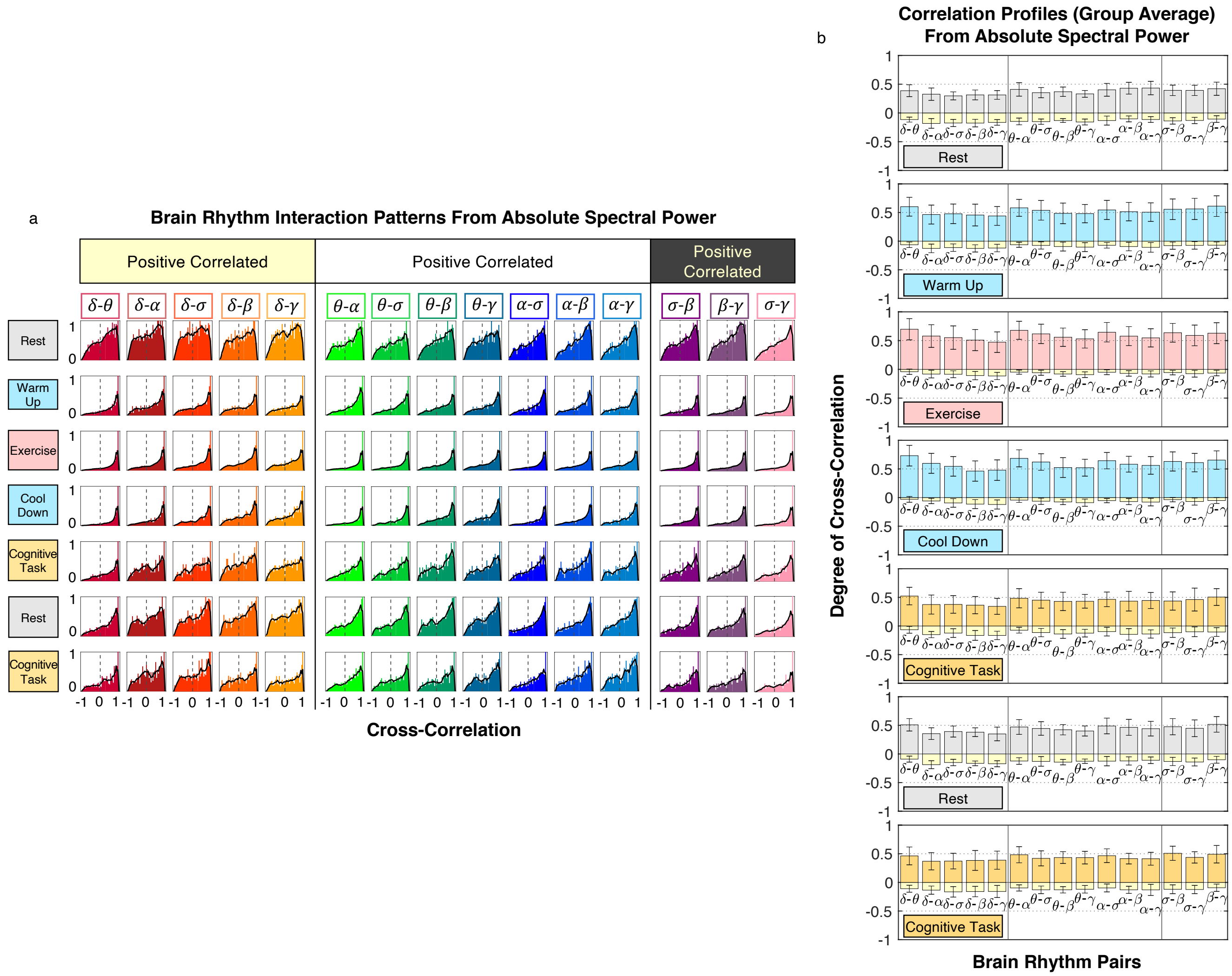

Supplementary Figure 8. Effect of relative versus absolute spectral power on assessing interactions between brain rhythms. a Group averaged interaction profiles for all pairs of brain rhythms at the C3 EEG channel location obtained using the absolute spectral power in their corresponding frequency bands (see Methods). Profiles represent rescaled histograms of cross-correlation values calculated in non-overlapping 30s windows pooled from all subjects (solid black lines show smoothed profiles by moving average). Due to global modulations in the EEG amplitude and correspondingly in the total EEG spectral power in response to changes in physiologic regulation and external factors (e.g., movement artifacts and scalp connectivity) all frequency bands are simultaneously affected in the same manner of increasing or decreasing their absolute power, leading to similar positive cross-correlation profiles for all pairs of brain rhythms across all physiological states. Thus, utilizing the absolute spectral power of brain rhythms leads to spurious effects due to common macro-trends, which mask the distinct forms of interaction among brain rhythms that underlie each physiological state (Figure 2 and Supplementary Figure 2). The test demonstrates that considering the relative spectral power is essential to quantify the micro-architecture of synchronized, short-time modulations in the amplitude of brain rhythms that occur on top of their quasi-steady-state behavior and large timescale trends, and give rise to different forms of coupling profiles. **b** Bar plots quantify the degree of cross-correlation for all pairs of brain rhythms obtained from the distribution profiles in (a), and show group averaged degree of coupling and standard deviations for each pair of brain rhythms. Results demonstrate strong positive cross-correlations with no differentiation between pairs for a given physiological state and similar pattern for all physiological states (no stratification) when the absolute spectral power for brain rhythms is used. In contrast, analyses of the relative spectral power of brain rhythms demonstrate three distinct classes of coupling forms and differentiated response of brain wave interactions to changes in physiologic state characterized by pronounced stratification (Figure 3 and Supplementary Figure 4).

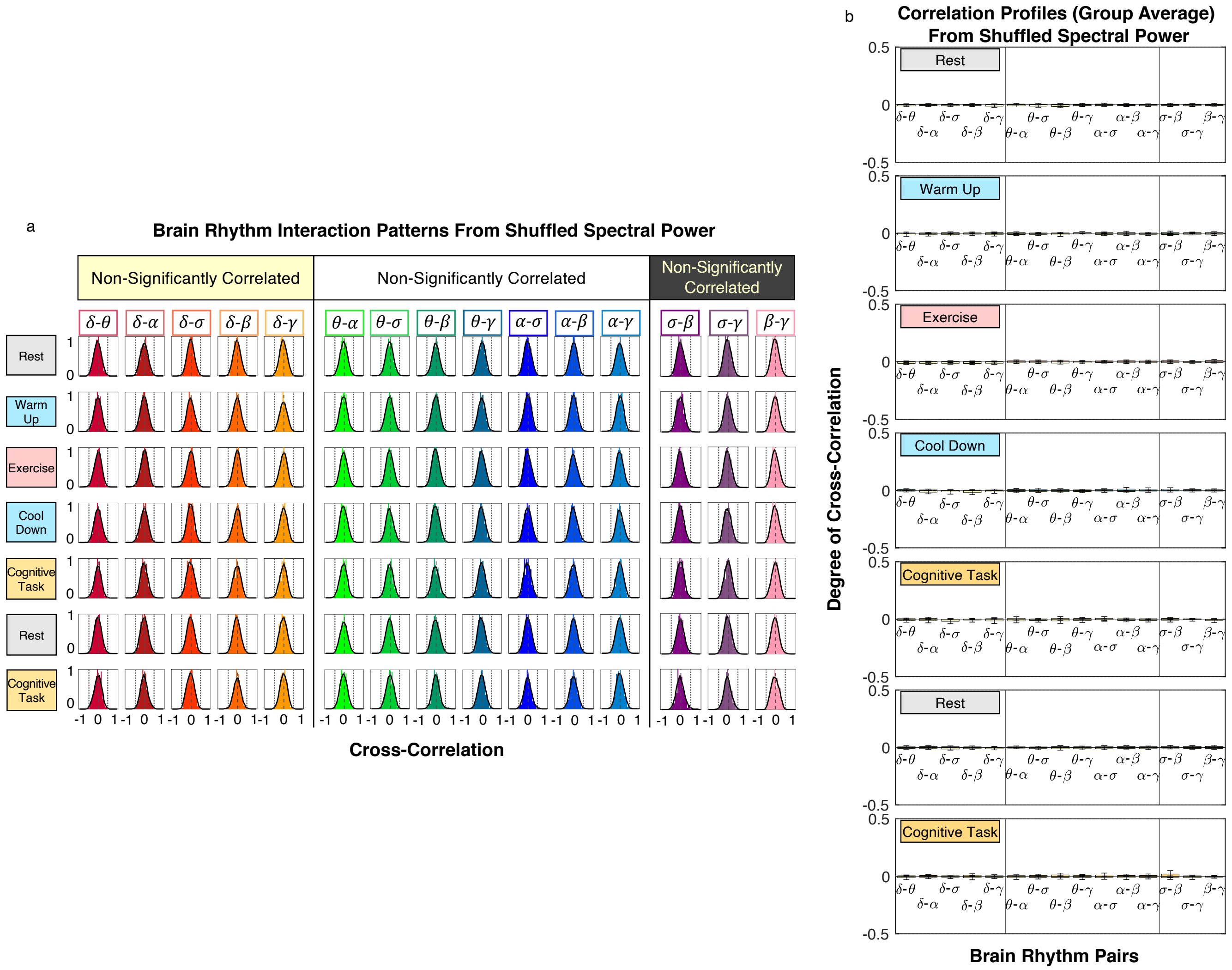

Supplementary Figure 9. Surrogate test based on shuffled data to define the significance threshold for cross-correlation values. a Cross-correlation distribution profiles from randomly shuffled time series of the relative spectral power of each brain rhythm (frequency band) at the C3 EEG channel location. Profiles are obtained by pooling randomized data from all subjects and exhibit close to gaussian shape. This surrogate test shows the effect of our analysis method on random signals derived from the original data, and establishes a minimum baseline for cross-correlation significance. Cross-correlation values are distributed around $C \approx 0$ with variance from non-overlapping 30s windows used in the analysis (i.e., 30 data points are used to calculate each cross-correlation value). The distribution profiles obtained for all surrogate pairs of frequency bands are practically identical for all physiological states, with vanishing probability at $C = \pm 0.5$ (vertical dotted lines in each panel), which defines the significance threshold for our analyses. The test indicates that the distinct classes of coupling profiles between brain rhythms and their stratification across physiological states obtained from empirical data (Figures 2 and 3, Supplementary Figures 2 and 4) reflect physiologically relevant interactions among brain rhythms. **b** Bar plots show group averages and standard deviations for the degree of cross-correlation derived from the surrogate test profiles in (a). The degree of cross-correlation is practically zero, indicating physiological significance of the results obtained from the empirical data.

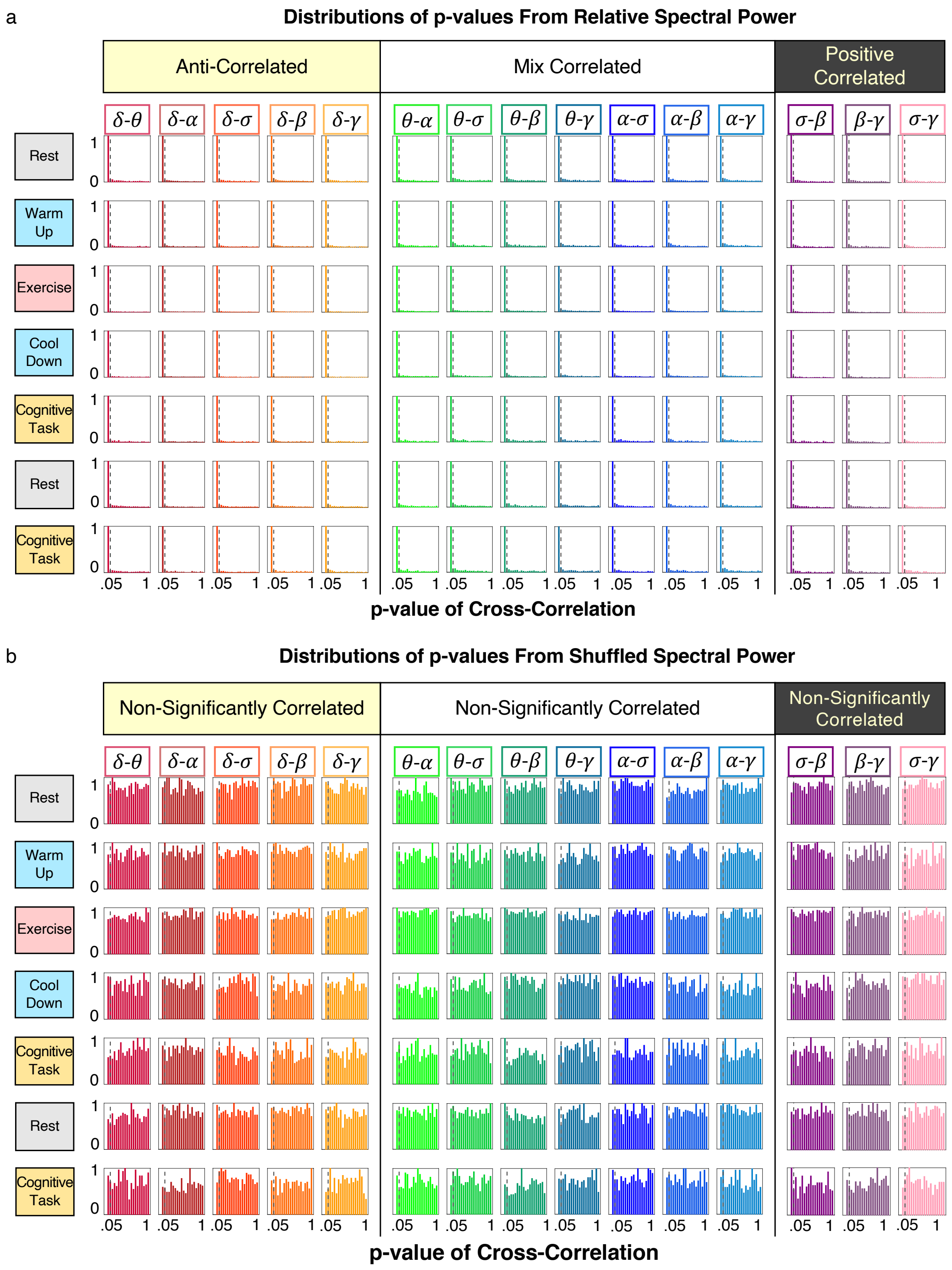

Supplementary Figure 10. Test for statistical significance of the distinct classes of brain wave interaction profiles. a Normalized distributions of p-values for Pearson cross-correlation coefficients obtained from empirical data and calculated in 30s windows from the C3 EEG recordings of all subjects. All histograms peak at $p < 0.05$ rejecting the null hypothesis that the analyzed data are random samples, thus confirming the statistical significance of the functional form of coupling profiles for all pairs of brain rhythms and the reported three distinct classes of brain wave interactions (positively correlated, anti-correlated and mixed correlated). **b** Normalized distributions of p-values for Pearson cross-correlation coefficients obtained in 30s windows from the C3 EEG recordings pooled from all subjects after shuffling the spectral power time series of paired brain rhythms (corresponding to the profiles from the surrogate shuffled data test shown in Supplementary Figure 9). In contrast to (a), the uniform distributions with $> 96\%$ of the p-values above $p = 0.05$ for all pairs of frequency bands and all physiological states confirm the null hypothesis that the analyzed data are random.

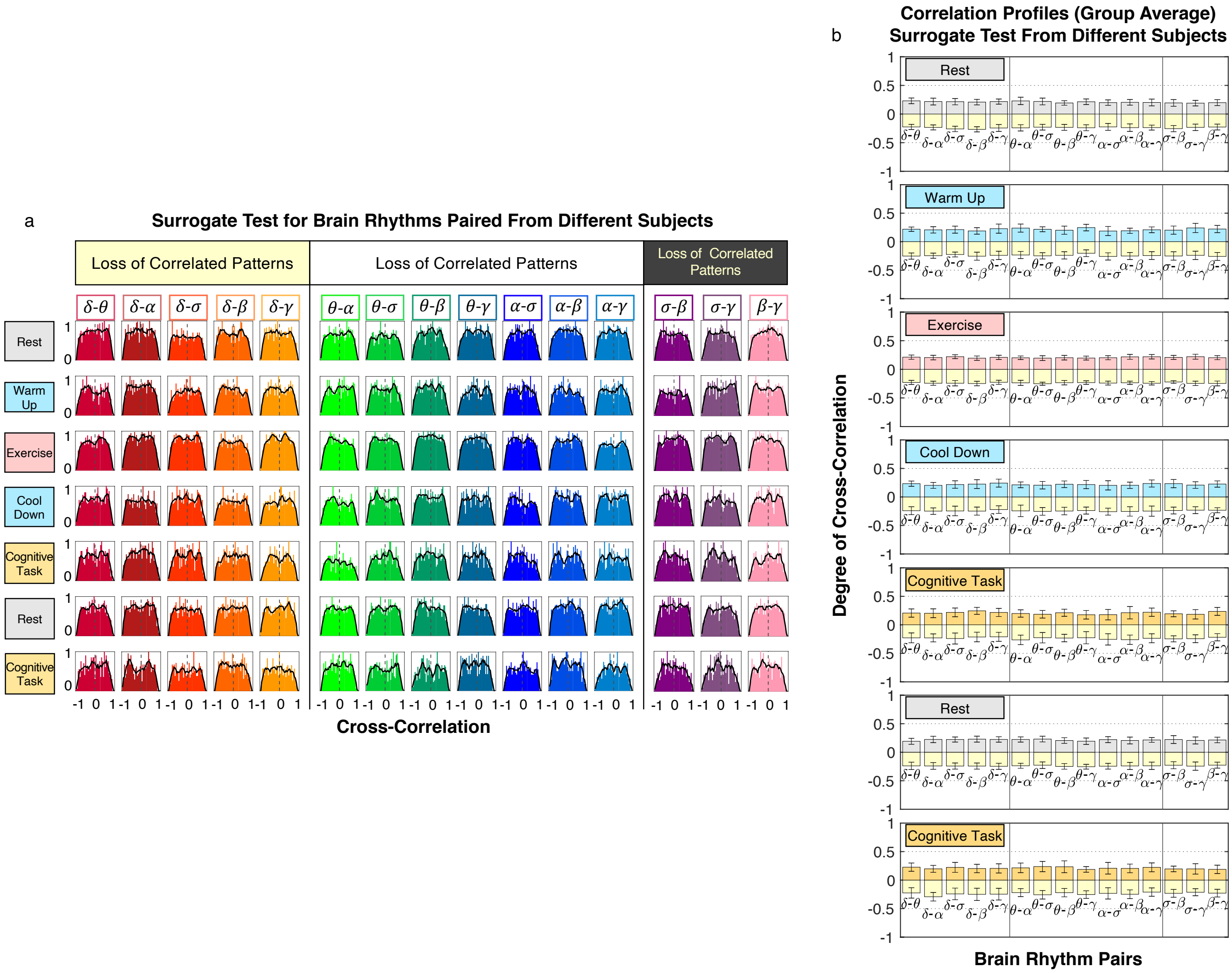

Supplementary Figure 11. Surrogate test where brain wave spectral power signals are randomly paired from two different subjects. a Distributions of Pearson cross-correlation coefficients for surrogate pairs representing each combination of brain rhythms in each physiological state. Each distribution profile is obtained by pooling together cross-correlation values in 30s windows from the spectral power time series of 19 surrogate pairs, where brain rhythms are paired from two different, randomly chosen subjects in the database – the 19 random realizations of surrogate pairs are selected to match the number of subjects in the database and the statistics of the surrogate test with the statistical power of the original results (Figures 2 and 3, Supplementary Figures 2 and 4). Uniform profiles for surrogate tests of pairing brain rhythms from different subjects indicate absence of consistent cross-correlations and coupling. **b** Bar plots represent the degree of cross-correlations derived from the distribution profiles in (a), showing no differentiation among different surrogate pairs of brain rhythms, equal amounts of positive and negative correlations, and no stratification with physiological states. While the original characteristics of the separate brain wave spectral power signals are preserved in this surrogate analysis, the test demonstrates that the uncovered distinct profiles of brain wave coupling and three major classes of interactions among brain rhythms reveal real physiological processes of synchronous modulation in the amplitudes of brain rhythms embedded at short timescales in the micro-architecture of brain dynamics.

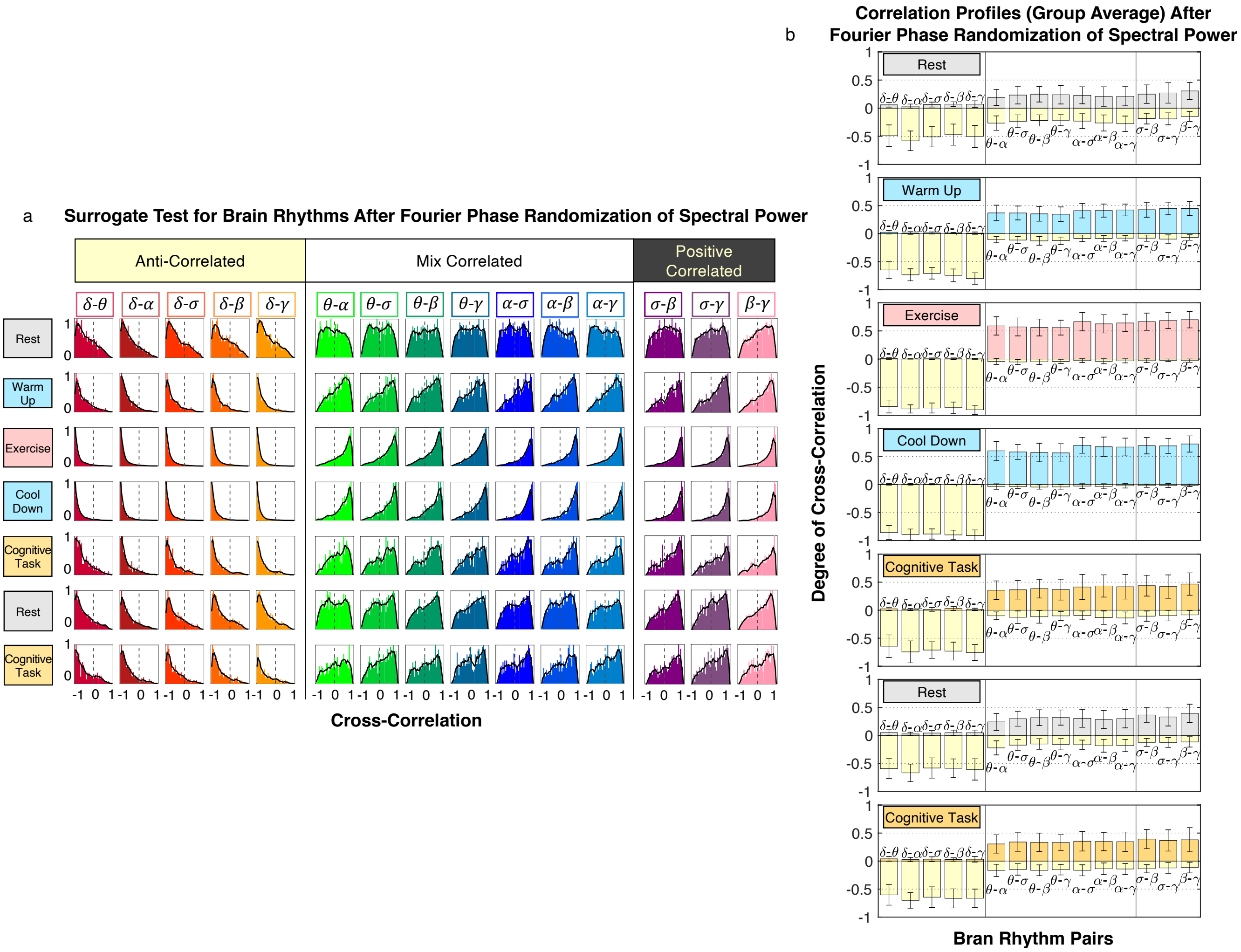

Supplementary Figure 12. **Surrogate test with Fourier Phase randomization of EEG signals. a** Interaction profiles from Fourier Phase randomization test performed for all pairs of brain rhythms during distinct physiological states. While the three classes of anti-correlated, positively correlated and mix correlated brain waves are still present, the shapes of the profiles and how the profiles evolve with transitions across physiological states differ from the real data. The surrogate test preserves the relative spectral power of the frequency bands in EEG signals corresponding to the different brain rhythms, but leads to temporal reorganization in the instantaneous amplitudes of brain rhythms, thus altering synchronous amplitude modulation between brain rhythms at short timescales and correspondingly affecting the functional form of coupling profiles. **b** Degree of cross-correlation for Fourier phase randomized surrogate data shows patterns different from the real data. The surrogate test demonstrates that the reported functional forms of brain wave coupling reflect physiological information related to both reorganization in the spectral power of different brain rhythms at large time scales as well as in their synchronous modulation at short time scales that occur in response to change in physiologic regulation during rest, exercise and cognitive task.

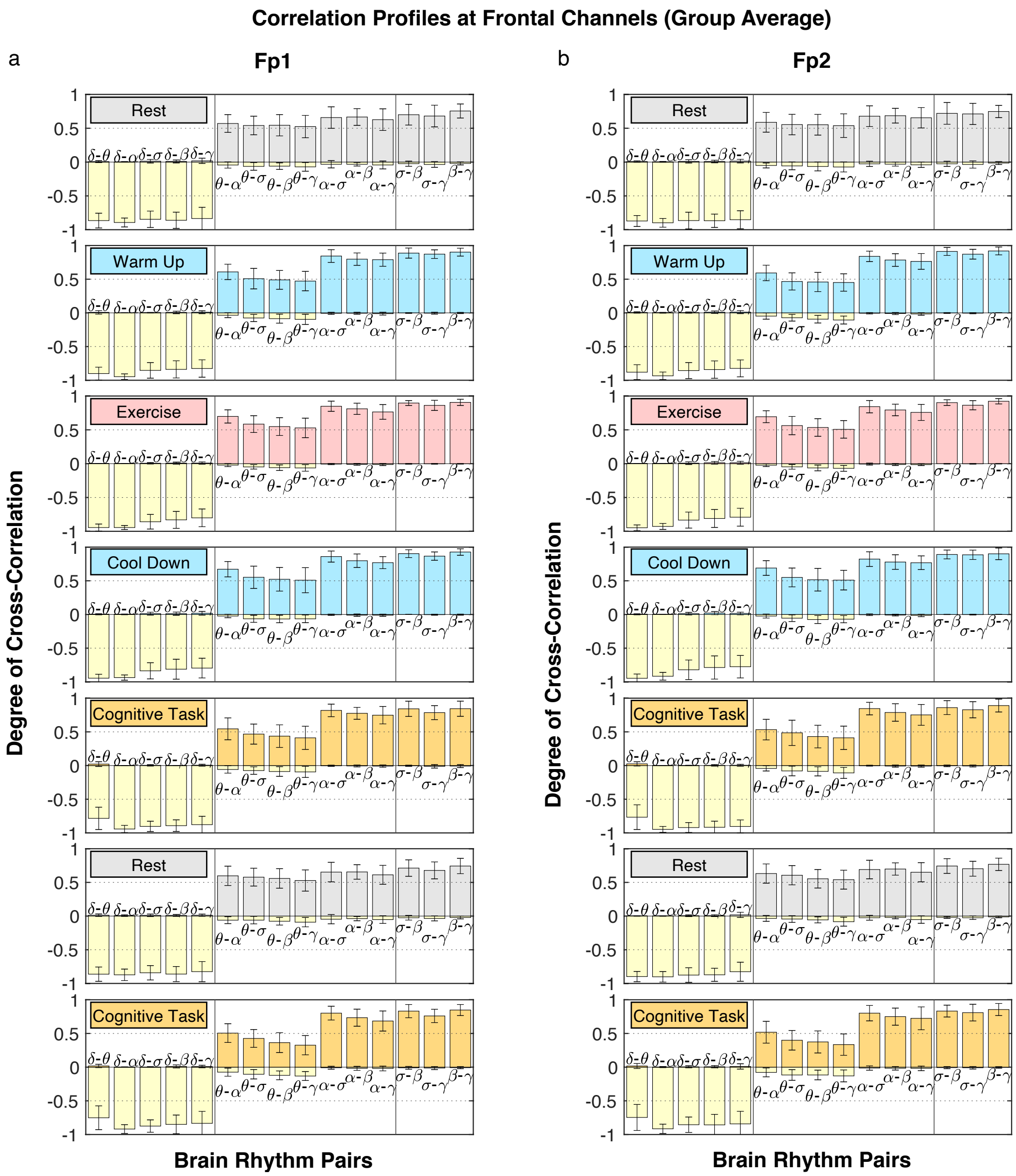

Supplementary Figure 13. Consistent patterns of brain wave interactions at frontal left and right hemisphere brain areas. Degree of coupling for all pairs of brain rhythms derived from frontal cortical areas Fp1 and Fp2 representing the (a) left and (b) right hemisphere. For each pair of brain rhythms, positive and negative bars represent the fraction of the recording during a specific physiologic state with significant positive correlation ($C > 0.5$) and significant anti-correlation ($C < -0.5$), respectively. Three major classes of brain wave interactions are clearly observed across physiological states for both (a) left and (b) right brain hemisphere, and the same stratification in brain rhythm interactions across physiological states is observed for both hemispheres.

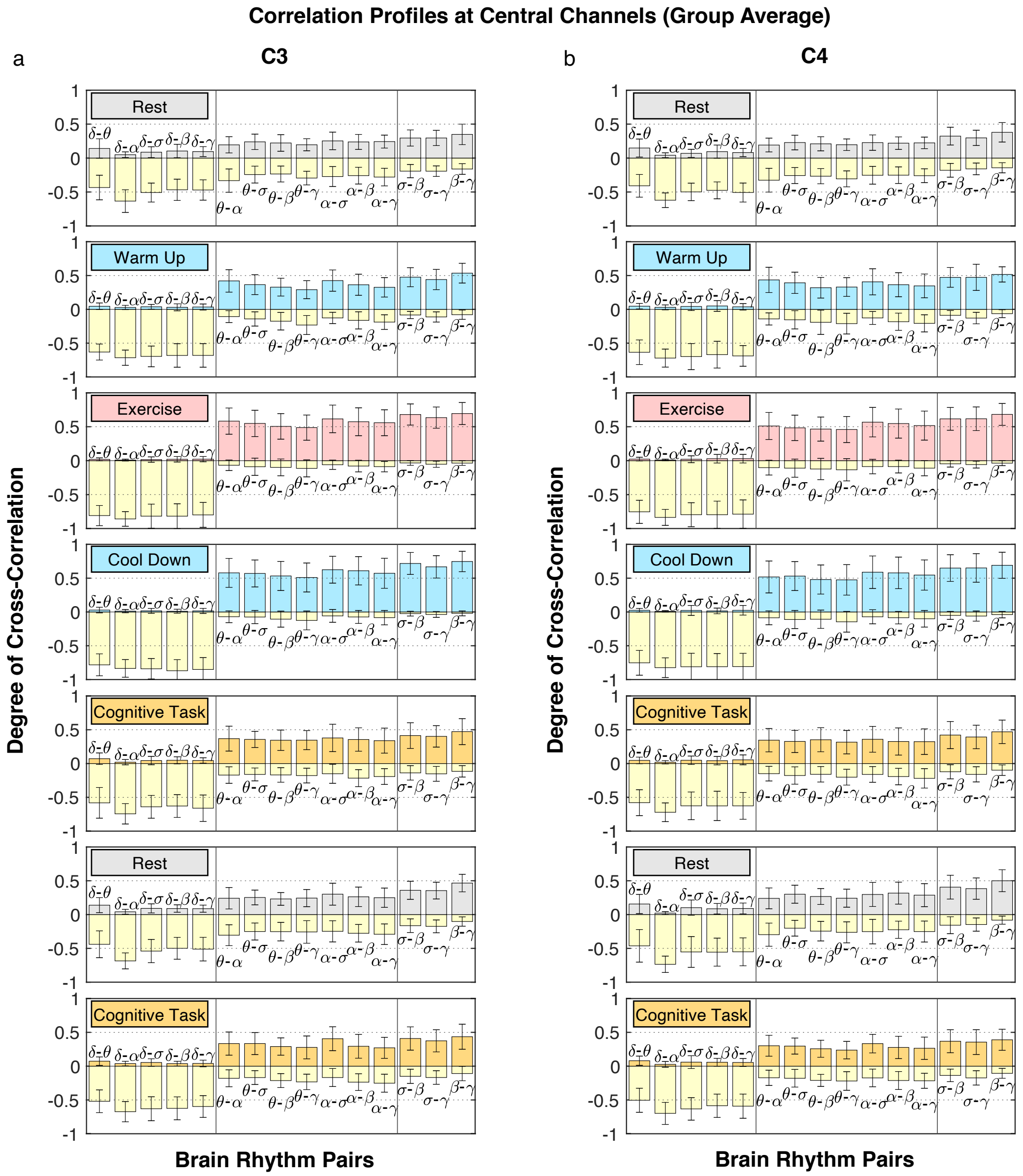

Supplementary Figure 14. Consistent patterns of brain wave interactions at central left and right hemisphere brain areas. Degree of coupling for all pairs of brain rhythms derived from central cortical areas C3 and C4 representing the (a) left and (b) right hemisphere. For each pair of brain rhythms, positive and negative bars represent the fraction of the recording during a specific physiologic state with significant positive correlation ($C > 0.5$) and significant anti-correlation ($C < -0.5$), respectively. Three major classes of brain wave interactions are clearly observed across physiological states for both (a) left and (b) right brain hemisphere, and the same stratification in brain rhythm interactions across physiological states is observed for both hemispheres.

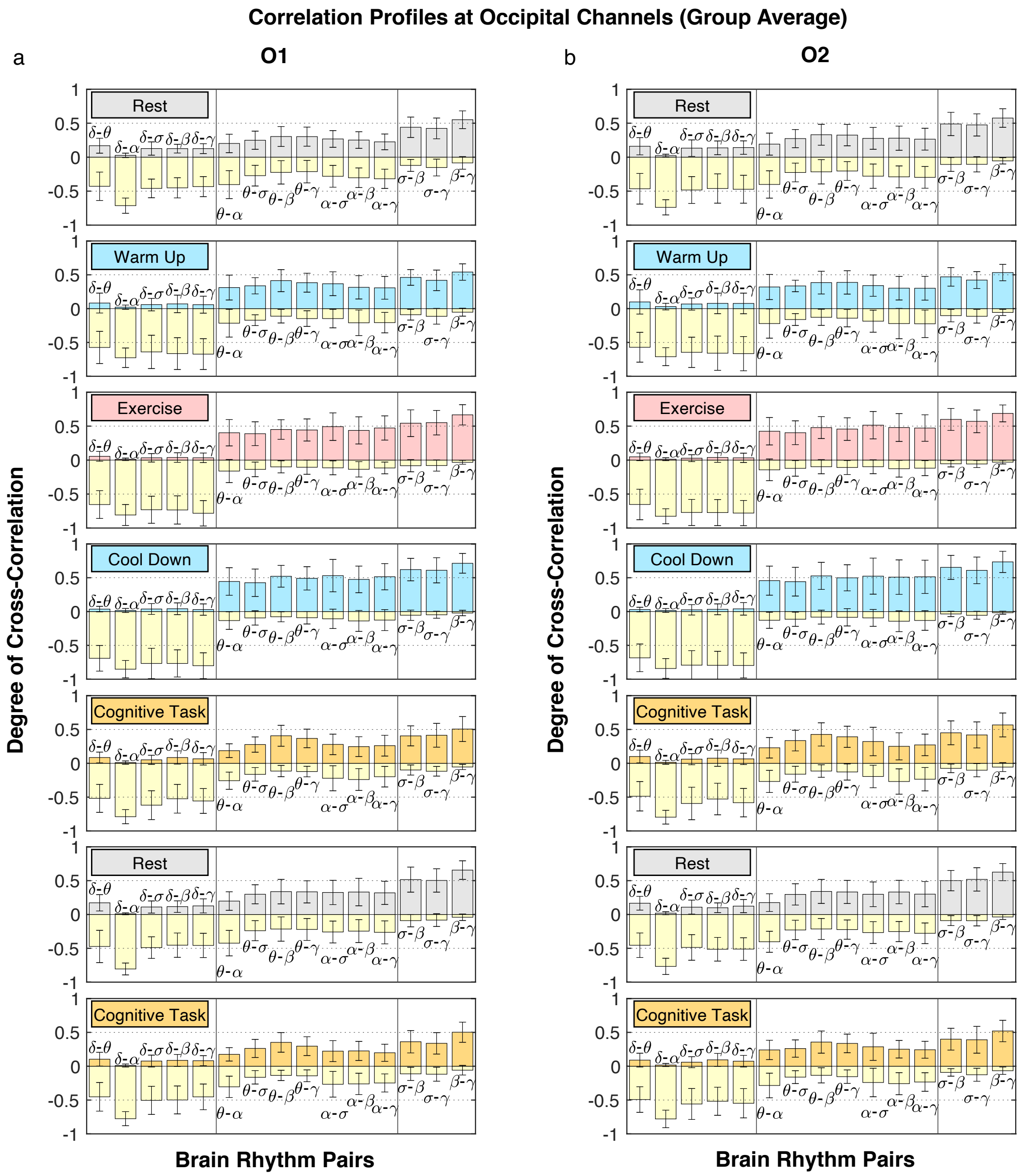

Supplementary Figure 15. Consistent patterns of brain wave interactions at occipital left and right hemisphere brain areas. Degree of coupling for all pairs of brain rhythms derived from occipital cortical areas O1 and O2 representing the (a) left and (b) right hemisphere. For each pair of brain rhythms, positive and negative bars represent the fraction of the recording during a specific physiologic state with significant positive correlation ($C > 0.5$) and significant anti-correlation ($C < -0.5$), respectively. Three major classes of brain wave interactions are clearly observed across physiological states for both (a) left and (b) right brain hemisphere, and the same stratification in brain rhythm interactions across physiological states is observed for both hemispheres. While brain rhythm interactions exhibit a clear symmetry between the left and right hemisphere for the frontal, central and occipital cortical areas (as shown in Supplementary Figures 13-15), there is gradual modulation in the strength of coupling for all pairs of brain rhythms with stronger positive and anti-correlated coupling (higher/lower bars) at the frontal area, intermediate at the central area and weaker coupling at the occipital area.
