# Supplementary material for: Ensemble of coupling forms and networks among brain rhythms as function of states and cognition
Source: Commun Biol. 2022 Jan 21;5:82. doi: 10.1038/s42003-022-03017-4 (PMC8782865; doi:10.1038/s42003-022-03017-4)
Supplement: Supplementary file 4 — Reporting Summary [file 42003_2022_3017_MOESM4_ESM.pdf]

nature research

Corresponding author(s): Plamen Ch. Ivanov

Last updated by author(s): Oct 29, 2021

Reporting Summary

Nature Research wishes to improve the reproducibility of the work that we publish. This form provides structure for consistency and transparency in reporting. For further information on Nature Research policies, see our Editorial Policies and the Editorial Policy Checklist.

Statistics

For all statistical analyses, confirm that the following items are present in the figure legend, table legend, main text, or Methods section.

n/a	Confirmed	
☐	☒	The exact sample size (n) for each experimental group/condition, given as a discrete number and unit of measurement
☐	☒	A statement on whether measurements were taken from distinct samples or whether the same sample was measured repeatedly
☐	☒	The statistical test(s) used AND whether they are one- or two-sided<br>*Only common tests should be described solely by name; describe more complex techniques in the Methods section.*
☒	☐	A description of all covariates tested
☐	☒	A description of any assumptions or corrections, such as tests of normality and adjustment for multiple comparisons
☐	☒	A full description of the statistical parameters including central tendency (e.g. means) or other basic estimates (e.g. regression coefficient) AND variation (e.g. standard deviation) or associated estimates of uncertainty (e.g. confidence intervals)
☐	☒	For null hypothesis testing, the test statistic (e.g. F, t, r) with confidence intervals, effect sizes, degrees of freedom and P value noted<br>*Give P values as exact values whenever suitable.*
☒	☐	For Bayesian analysis, information on the choice of priors and Markov chain Monte Carlo settings
☒	☐	For hierarchical and complex designs, identification of the appropriate level for tests and full reporting of outcomes
☐	☒	Estimates of effect sizes (e.g. Cohen's d, Pearson's r), indicating how they were calculated

Our web collection on statistics for biologists contains articles on many of the points above.

Software and code

Policy information about availability of computer code

Data collection	We used the BIOPAC system and software for EEG data acquisition, collected from the 30-channel actiCHamp System (Brain Products GmbH, Munich, Germany). We used a ViaSprint 150P cycle ergometer (Ergoline GmbH, Germany) to induce physical effort, obtain pedaling power values, and a JAEGER Master Screen gas analyzer (CareFusion GmbH, Germany) to measure gas exchange during the test.
Data analysis	We used MATLAB (2021b) and the EEGLAB software package to analyze the data and to perform statistical tests.

For manuscripts utilizing custom algorithms or software that are central to the research but not yet described in published literature, software must be made available to editors and reviewers. We strongly encourage code deposition in a community repository (e.g. GitHub). See the Nature Research guidelines for submitting code & software for further information.

Data

Policy information about availability of data

All manuscripts must include a data availability statement. This statement should provide the following information, where applicable:

- Accession codes, unique identifiers, or web links for publicly available datasets
- A list of figures that have associated raw data
- A description of any restrictions on data availability

The data analyzed in this work are multi-channel EEG recordings from the REXCO Project (http://doi.org/10.5281/zenodo.1237654).

Field-specific reporting

Please select the one below that is the best fit for your research. If you are not sure, read the appropriate sections before making your selection.

☒ Life sciences ☐ Behavioural & social sciences ☐ Ecological, evolutionary & environmental sciences

For a reference copy of the document with all sections, see nature.com/documents/nr-reporting-summary-flat.pdf

Life sciences study design

All studies must disclose on these points even when the disclosure is negative.

Sample size	High-frequency (1,000Hz) EEG data were collected from 19 subjects, continuously over 120 minutes and analyzed in short time-segments of 30 seconds, yielding 4,560 data points for identification of cross-correlation profiles and coupling strengths/degree of synchronization between cortical rhythms as a function of different physiological states (which is approximately two magnitudes higher than necessary for comparative statistical tests based on null-hypothesis).
Data exclusions	20 healthy subjects participated in the experiment; analyses are based on data from 19 subjects (1 subject, out of 20, was excluded because he failed to complete all experimental protocols).
Replication	Each of the 19 subjects performed two similar experimental protocol tests (120 min each) at two different physical effort levels, while keeping the rest and cognitive task conditions identical. Within each experimental test, each subject performed two repeated sections for rest and two for cognitive task to confirm the consistency of results. The two tests were separated by at least 48 hours and no more than 72 hours to avoid possible fatigue or training effects. Each participant attended both tests at the same time of the day to avoid circadian effects on the level of test performance. For details, see Methods Section.
Randomization	N/A. Only healthy subjects were analyzed.
Blinding	N/A. Only healthy subjects were analyzed.

Reporting for specific materials, systems and methods

We require information from authors about some types of materials, experimental systems and methods used in many studies. Here, indicate whether each material, system or method listed is relevant to your study. If you are not sure if a list item applies to your research, read the appropriate section before selecting a response.

Materials & experimental systems

n/a	Involved in the study	
☒	☐	Antibodies
☒	☐	Eukaryotic cell lines
☒	☐	Palaeontology and archaeology
☒	☐	Animals and other organisms
☐	☒	Human research participants
☒	☐	Clinical data
☒	☐	Dual use research of concern

Methods

n/a	Involved in the study	
☒	☐	ChIP-seq
☒	☐	Flow cytometry
☒	☐	MRI-based neuroimaging

Human research participants

Policy information about studies involving human research participants

Population characteristics	20 young males, age 19-32 years (average 23.8 yrs), were recruited from a pool of undergraduate students from the University of Granada, Spain.
Recruitment	Participants met inclusion criteria of: reporting less than 3 hours of moderate physical activity per week; normal or corrected to normal vision; no neurological, cardiovascular, or musculoskeletal disorders; no medication in-take. No invasive procedures were involved in the experimental protocol.
Ethics oversight	The protocol was approved following the University of Granada's ethical guidelines and the Declaration of Helsinki of 1964. All subjects gave written informed consent before the study.

Note that full information on the approval of the study protocol must also be provided in the manuscript.
